# NR2F1 shapes mitochondria in the mouse brain, providing new insights into Bosch-Boonstra-Schaaf optic atrophy syndrome

**DOI:** 10.1242/dmm.049854

**Published:** 2023-06-26

**Authors:** Sara Bonzano, Eleonora Dallorto, Ivan Molineris, Filippo Michelon, Isabella Crisci, Giovanna Gambarotta, Francesco Neri, Salvatore Oliviero, Ruth Beckervordersandforth, Dieter Chichung Lie, Paolo Peretto, Serena Bovetti, Michèle Studer, Silvia De Marchis

**Affiliations:** ^1^ Department of Life Sciences and Systems Biology (DBIOS), University of Turin, Via Accademia Albertina 13, Turin 10123, Italy; ^2^ Neuroscience Institute Cavalieri Ottolenghi (NICO), Regione Gonzole 10, Orbassano 10043, Italy; ^3^ IIGM Foundation-Italian Institute for Genomic Medicine, Sp142 Km 3.95, Candiolo 10060, Italy; ^4^ Department of Clinical and Biological Sciences (DSCB), Regione Gonzole 10, Orbassano 10043, Italy; ^5^ Institut für Biochemie, Friedrich-Alexander Universität Erlangen-Nürnberg (FAU), Fahrstrasse 17, Erlangen 91054, Germany; ^6^ Institute de Biologie Valrose (iBV), Université Côte d'Azur (UCA), CNRS 7277, Inserm 1091, Avenue Valrose 28, Nice 06108, France

**Keywords:** COUP-TFI, Mitochondrial dysfunction, Dentate gyrus, Intellectual disability, Adult-born neurons, BBSOAS

## Abstract

The nuclear receptor NR2F1 acts as a strong transcriptional regulator in embryonic and postnatal neural cells. In humans, mutations in the *NR2F1* gene cause Bosch-Boonstra-Schaaf optic atrophy syndrome (BBSOAS), a rare neurodevelopmental disorder characterized by multiple clinical features including vision impairment, intellectual disability and autistic traits. In this study, we identified, by genome-wide and *in silico* analyses, a set of nuclear-encoded mitochondrial genes as potential genomic targets under direct NR2F1 transcriptional control in neurons. By combining mouse genetic, neuroanatomical and imaging approaches, we demonstrated that conditional NR2F1 loss of function within the adult mouse hippocampal neurogenic niche results in a reduced mitochondrial mass associated with mitochondrial fragmentation and downregulation of key mitochondrial proteins in newborn neurons, the genesis, survival and functional integration of which are impaired. Importantly, we also found dysregulation of several nuclear-encoded mitochondrial genes and downregulation of key mitochondrial proteins in the brain of *Nr2f1*-heterozygous mice, a validated BBSOAS model. Our data point to an active role for NR2F1 in the mitochondrial gene expression regulatory network in neurons and support the involvement of mitochondrial dysfunction in BBSOAS pathogenesis.

## Teaser

**Summary:** Loss of NR2F1, which regulates mitochondrial genes in neurons, leads to mitochondrial dysfunction and impaired neuron function, which could contribute to pathogenesis of the neurodevelopmental disorder Bosch-Boonstra-Schaaf optic atrophy syndrome.

## INTRODUCTION

The transcriptional regulator nuclear receptor subfamily 2, group f, member 1 (NR2F1), also known as chicken ovalbumin upstream promoter-transcription factor 1 (COUP-TFI), has been extensively investigated in brain development, during which it exerts pleiotropic functions, including the control of neural stem cell competency, axonal pathfinding, cortical area and cell-type specification in mice (Naka et al., 2008; Alfano et al., 2014; Armentano et al., 2006; reviewed in Bertacchi et al., 2019b). Its expression is maintained in the postnatal and adult brain, in which it controls activity-dependent expression of tyrosine hydroxylase in olfactory dopaminergic neurons (Bovetti et al., 2013) and regulates neuron–astroglia cell fate decision in the neurogenic niche of the hippocampal dentate gyrus (DG) (Bonzano et al., 2018).

In humans, *NR2F1* has recently emerged as a disease gene: multiple *NR2F1* pathological variants cause Bosch-Boonstra-Schaaf optic atrophy syndrome [BBSOAS; Online Mendelian Inheritance in Man (OMIM) 615722], a rare, monogenic autosomal-dominant disorder characterized by multiple clinical features, including global developmental delay, mild-to-severe intellectual disability (ID), optic nerve atrophy, vision impairments, seizures and traits characteristic of autism spectrum disorder (Bosch et al., 2014; Chen et al., 2016; Kaiwar et al., 2017). Most variants in the *NR2F1* gene of patients described so far are deletions and/or mutations predominantly located in the DNA-binding domain and lead to haploinsufficiency or dominant-negative effects, thus compromising and/or completely abolishing NR2F1 transcriptional regulatory activity (Billiet et al., 2021; Bosch et al., 2016; Chen et al., 2016; Kaiwar et al., 2017; Rech et al., 2020). Several neurological symptoms – such as optic atrophy, hypotonia and seizure – described in BBSOAS patients, are often associated with mitochondrial dysfunction in the nervous system (Frye, 2020; Lenaers et al., 2021; Valenti et al., 2014; Zsurka and Kunz, 2015). Nevertheless, no data are available on possible mitochondrial implications in the neurological symptoms of BBSOAS patients, and whether and how NR2F1 can affect mitochondria in neuronal cells is still unknown.

Mitochondria are multi-functional and highly dynamic organelles, essential for neuronal development and function. For instance, dendritogenesis, axon outgrowth and neurite architecture are directly influenced by regulated transport, fusion–fission, and anchoring of mitochondria (Rangaraju et al., 2019b; Misgeld and Schwarz, 2017). Moreover, in mature neurons, mitochondria contribute to synaptic transmission and plasticity by providing local energy supply and Ca^2+^ buffering (Rangaraju et al., 2019a). The regulation of mitochondrial biogenesis, dynamics and function is under a transcriptional network including DNA-binding factors that target nuclear [e.g. NRF1, NRF2 (NFE2L2), PPARs, ERRs (ESRRs)] or mitochondrial DNA (e.g. TFAM, TFB1M/TFB2M). Additionally, this network includes co-regulators that integrate signals and coordinate activities of multiple DNA-binding factors in a tissue- and signal-specific expression pattern (Hock and Kralli, 2009).

In this study, we identified, for the first time, several nuclear-encoded mitochondrial genes among the genomic targets under direct NR2F1 transcriptional regulation in neurons by genome-wide and *in silico* analyses. To directly assess whether NR2F1 controls mitochondria, we took advantage of mice heterozygous for *Nr2f1*, a validated BBSOAS mouse model (Bertacchi et al., 2019a; Jurkute et al., 2021), in which we unveiled altered expression of several mitochondrial genes associated with decreased levels of key mitochondrial proteins. Furthermore, we exploited the Cre-loxP model to conditionally manipulate *Nr2f1* in adult neural stem/progenitor cells (aNSPCs) (Bonzano et al., 2018) and studied the effects on their neuronal lineage. aNSPCs persist throughout life in restricted brain regions, namely the subventricular zone of the lateral ventricles and the subgranular zone (SGZ) of the hippocampal DG (Bond et al., 2015). In the SGZ, aNSPCs ensure the continuous generation of new neurons, the integration of which into the pre-existing circuitries is crucial for proper adaptive behaviors and cognitive functions (Aimone et al., 2014). Interestingly, immature DG neurons show higher NR2F1 expression (Artegiani et al., 2017; Bonzano et al., 2018) and an overall increase in mitochondrial mass compared to that in aNSPCs (Beckervordersandforth et al., 2017; Steib et al., 2014). Moreover, mitochondrial dysfunction impairs adult DG neurogenesis (Beckervordersandforth et al., 2017; Beckervordersandforth, 2017; Khacho et al., 2016; Steib et al., 2014). Thus, we focused on adult SGZ neurogenesis as a suitable model to dissect the cell-intrinsic role of NR2F1 in mitochondria. Our findings demonstrate that the loss of NR2F1 function impairs the mitochondrial mass and shape as well as the oxidative phosphorylation (OxPhos) system of adult-born DG neurons. In addition, it leads to a reduction in their morphological complexity, functional integration and long-term survival. Importantly, our data provide the first demonstration of a role for NR2F1 in shaping neuronal mitochondria in the adult brain. This paves the way for further research on the mechanisms and role of mitochondrial dysfunction in the pathogenesis of BBSOAS, opening up new possibilities for therapeutic intervention.

## RESULTS

### NR2F1 nuclear genomic targets are enriched in key mitochondrial factors

**Fig. 1.**
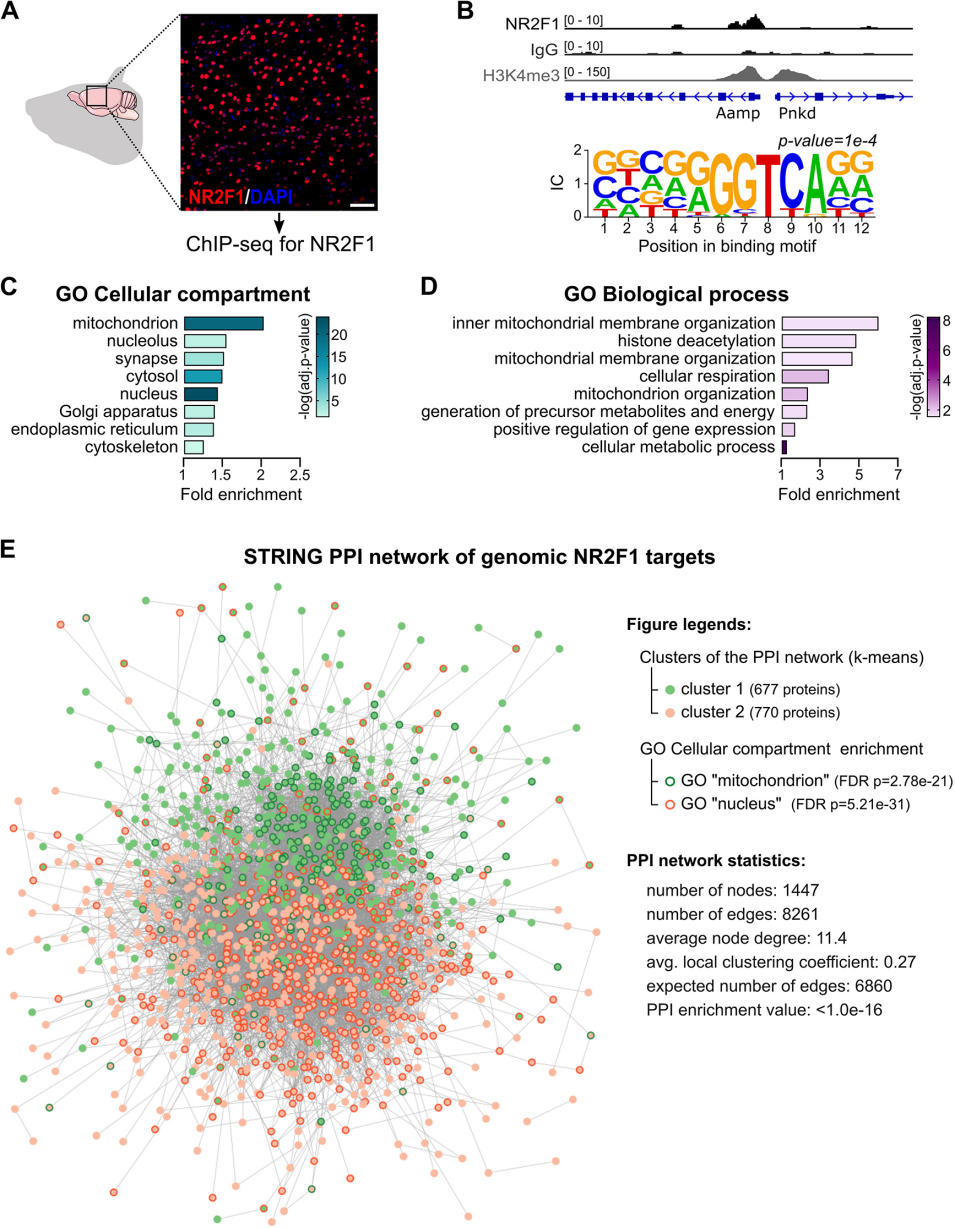
**Chromatin immunoprecipitation followed by deep sequencing (ChIP-seq) analysis of adult mouse neocortex revealed enrichment of mitochondrial proteins among NR2F1 genomic targets.** (A) ChIP-seq for NR2F1 was carried out on the adult mouse neocortex, a brain region enriched in neurons expressing high levels of NR2F1. Representative image of NR2F1 staining (red) in the adult mouse neocortical region. Nuclei are counterstained with DAPI (blue). Scale bar: 50[Supplementary-material sup1]μm. (B) Top: example of a genomic peak on a promoter showing NR2F1 binding associated with H3K4me3 (*Aamp* gene) compared with a promoter region showing H3K4me3 without NR2F1 binding (*Pnkd* gene). Bottom: NR2F1 binding sites (forward) revealed by *de novo* transcription factor motif discovery – see Fig. S1D for best matches to known motifs. IC, information content. (C,D) Bar graphs illustrating the fold enrichment by annotation with Gene Ontology (GO) terms, wherein the 2119 genes bound by NR2F1 were ranked for cellular components (C) and biological processes (D). Colors of the bars indicate the −log_10_(FDR) of the GO annotation term. The full lists of enriched categories are provided in Table S1. FDR, false discovery rate. (E) Protein–protein interaction (PPI) graph of the NR2F1 target genes identified by ChIP-seq and analyzed by STRING. Nodes are filled in either green or red colors based on a *k*-means clustering solution with *k*=2. These two clusters also segregate genes with GO enrichment terms in either the mitochondria or nuclear cell compartments (border colors of the node in green or red, respectively).

To investigate whether the nuclear transcription factor NR2F1 impinges on mitochondria by directly binding and regulating the expression of nuclear-encoded mitochondrial genes that code for more than 95% of the mitochondrial proteins (Calvo et al., 2016; Misgeld and Schwarz, 2017), we ran a genome-wide analysis of NR2F1 occupancy by chromatin immunoprecipitation followed by deep sequencing (ChIP-seq) in the adult neocortex, a brain region in which NR2F1-expressing neurons are abundant (Fig. 1A; Fig. S1A). By peak calling analysis, we identified 2119 binding sites for NR2F1 enriched in CpG islands and promoter regions [i.e. −3 kb/+2 kb from the transcription start site (TSS) of annotated genes] (Fig. S1B,C). Almost all NR2F1-bound promoters were positive for H3K4me3, a histone mark highly enriched at active promoters (Liang et al., 2004), but not all H3K4me3^+^ promoters were bound by NR2F1 (Fig. 1B), implying specificity. Notably, predicting transcription factor binding by the Homer motif discovery tool confirmed that the observed genomic peaks are highly enriched in the putative NR2F1 consensus sequence (Fig. 1B; Fig. S1D), indicating direct binding. The set of genes identified as putative NR2F1 genomic targets by ChIP-seq was then analyzed by Gene Ontology (GO) term annotation through the PANTHER classification system. Concerning the ‘cellular component’ class, the analysis revealed that the mitochondrion is the most enriched compartment (fold enrichment, >2), followed by other compartments showing fold enrichment of ∼1.5, including synapse and nucleus (Fig. 1C). In line, analysis of the ‘biological process’ class revealed enrichment of genes involved in mitochondrial function, particularly in the organization of the inner mitochondrial membrane (fold enrichment, >6) as well as in mitochondria-related metabolism and cellular respiration (Fig. 1D). Furthermore, the investigation of functional and physical protein associations among NR2F1 target genes using STRING analysis (Szklarczyk et al., 2021) showed more interactions among NR2F1 targets than expected for a random set of proteins of the same size and degree distribution [protein–protein interaction (PPI) enrichment *P*-value <1.0×10^−16^]. Interestingly, the interaction network was characterized by two clusters, the first enriched in mitochondrial proteins and the second enriched in nuclear proteins (Fig. 1E). This indicates that NR2F1 orchestrates a complex biological program involving several genes that operate in a highly interconnected way, and suggests that it may have a direct regulatory role in a wide set of nuclear-encoded mitochondrial genes in neurons.

### NR2F1 manipulations in adult-born hippocampal neurons lead to altered mitochondrial mass and morphology

**Fig. 2.**
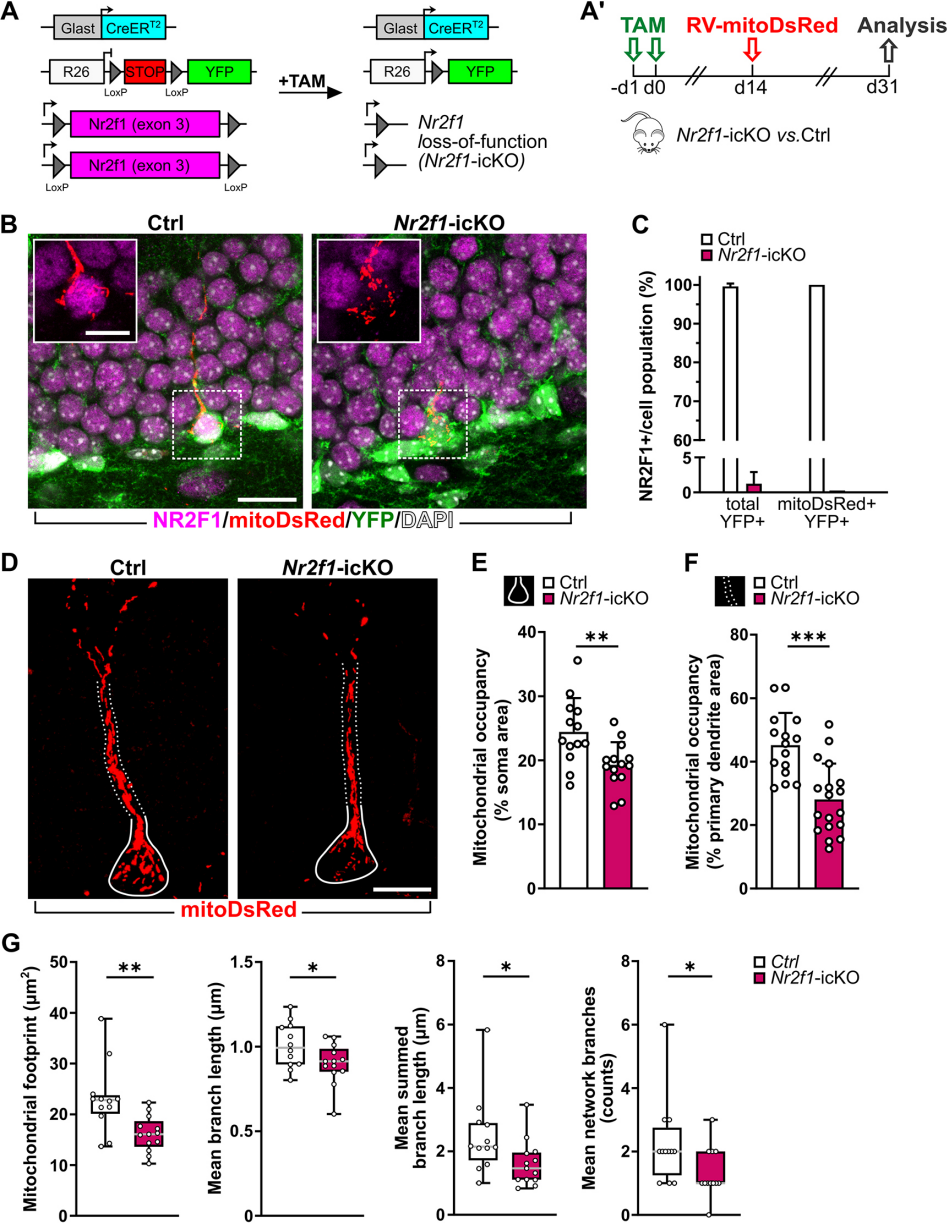
**NR2F1 loss of function leads to decreased mitochondrial mass in the soma and proximal dendritic compartment of adult-born hippocampal neurons *in vivo*.** (A) Overview of the Cre-mediated gene rearrangements of Rosa26 (R26) and *Nr2f1* loci in *Glast::CreERT2;R26-floxed STOP-Nr2f ^fl/fl^* mice. (A′) Experimental design. d, day; TAM, tamoxifen. (B) Representative confocal images of adult-born neurons transduced with RV-mitoDsRed (red) and immunostained for YFP (green) and NR2F1 (magenta) in Ctrl and *Nr2f1*-icKO mice. Nuclei are counterstained with DAPI (white). (C) Validation of NR2F1 loss in the Glast lineage. Whereas in control dentate gyrus (DG), the vast majority of YFP^+^ and mitoDsRed^+^YFP^+^ cells showed nuclei that are positive for NR2F1, in mutant DG, virtually all YFP^+^ and mitoDsRed^+^YFP^+^ cells are negative for NR2F1. NR2F1^+^ of total YFP^+^ cells: *n*=229/230 cells from three control (Ctrl) animals; *n*=5/340 cells from three *Nr2f1*-icKO animals. Nr2f1^+^ in the subset of mitoDsRed^+^YFP^+^ cells: *n*=16/16 cells from three Ctrl animals; *n*=0/20 cells from three *Nr2f1*-icKO animals. (D) Representative confocal images (maximum projection) showing the mitoDsRed^+^ mitochondria (red) within the soma area (solid lines) and primary dendrite (dotted lines) in control and *Nr2f1*-icKO mitoDsRed^+^DCX^+^YFP^+^ newborn neurons. (E,F) Mitochondrial occupancy expressed as the percentage of the soma area (E) or of the primary dendrite area (F) covered by the mitoDsRed signal. Mann–Whitney test; *P*=0.0066 (E); *P*=0.0001 (F). For the analyses reported in E and F, *n*=13 (Ctrl) and *n*=16 (*Nr2f1*-icKO), and *n*=14 (Ctrl) and *n*=18 (*Nr2f1*-icKO) cells, respectively, from three animals per genotype and at least three cells/animal were used. (G) Results obtained by applying the MiNA toolkit on the mitoDsRed signal within the soma compartment of control and *Nr2f1*-icKO newborn mitoDsRed^+^DCX^+^YFP^+^ neurons. From left to right: two-tailed unpaired Student's *t*-test with Welch's correction, **P=0.0061; two-tailed unpaired Student's *t*-test, *P=0.0465; Mann–Whitney test, *P=0.0248; Mann–Whitney test, *P=0.0341. A coherent reduction in the parameter of mitochondrial footprint (i.e. the total area consumed by mitochondrial signal after being separated from the background) with a coincident decrease in the length of branches (i.e. mean length of all the lines used to represent the mitochondrial structures) and in the summed branch length (i.e. the mean of the sum of the lengths of branches for each independent mitochondrial structure divided by the number of independent mitochondrial structures), as well as in the number of network branches (i.e. the mean number of attached lines used to represent each mitochondrial structure), was found in *Nr2f1*-icKO neurons compared to Ctrl neurons. Data are shown as mean±s.d. (C,E,F); box and whiskers plots (G) with median (middle gray line), upper/lower quartiles, and error bars ranging from minimum to maximum values. Each dot represents a cell. Scale bars: B, 20 µm; B (inset), 10 µm; D, 10 µm.

To study the role of NR2F1 in mitochondria, we focused on the neurogenic niche of the adult hippocampus. Our previous research has demonstrated that NR2F1 is widely expressed throughout the aNSPCs and neurogenic lineage of the hippocampal DG, and that the loss of NR2F1 in aNSPCs leads to impaired DG neurogenesis (Bonzano et al., 2018). Thus, we exploited an *in vivo* conditional loss-of-function (LOF) approach targeting the aNSPC lineage via tamoxifen (TAM) administration in triple transgenic mice carrying inducible Cre-recombinase (*CreERT2*) under the Glast promoter, both *Nr2f1* alleles flanked by loxP sites and the reporter gene encoding the yellow fluorescent protein (YFP) for lineage tracing (herein named *Nr2f1*-icKO; Fig. 2A) (Armentano et al., 2007; Bonzano et al., 2018). Animals carrying both *Cre* and YFP alleles but wild type (WT) for *Nr2f1* were used as controls (Ctrl).

**Fig. 3.**
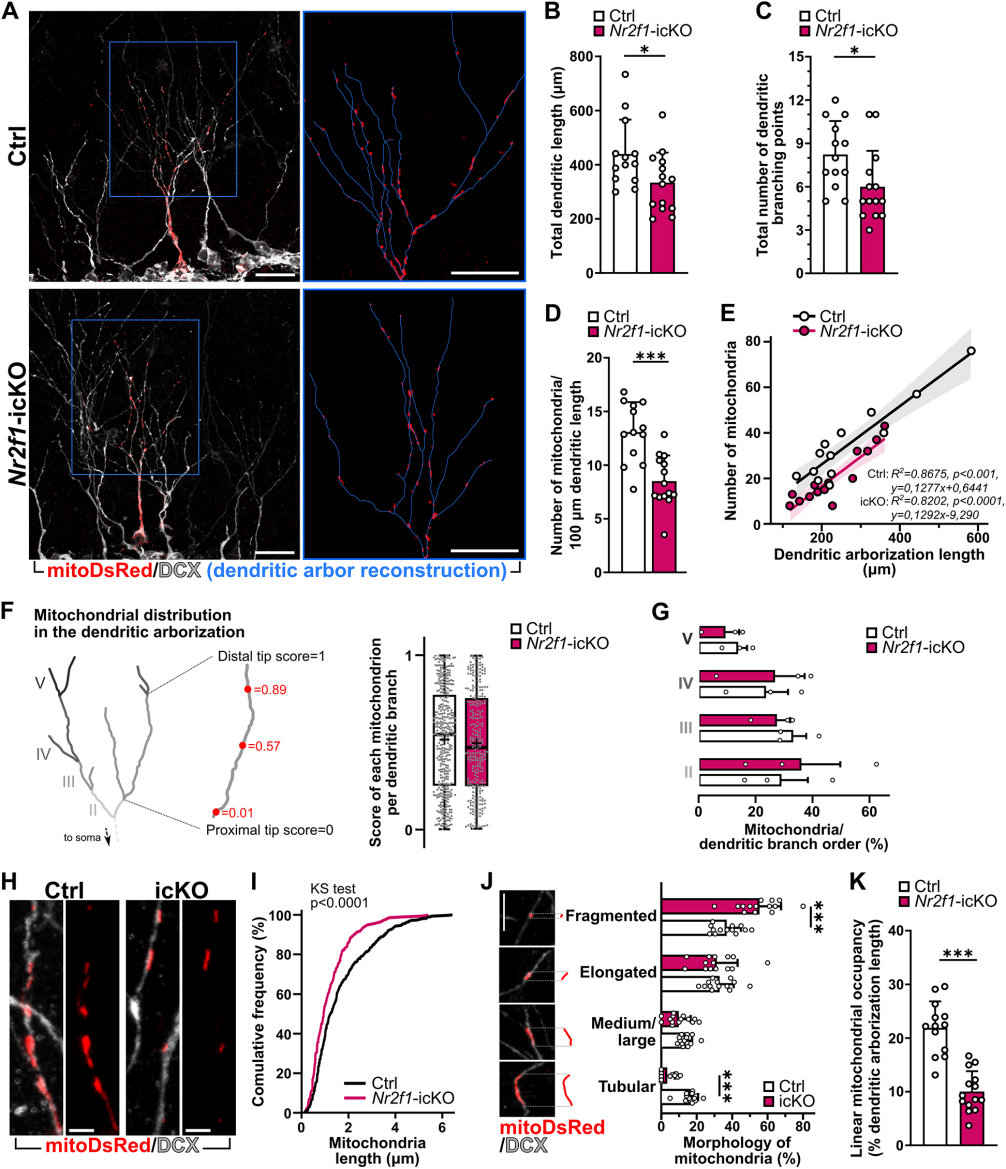
**Alteration in dendritic mitochondrial number, architecture and mass in newborn neurons lacking NR2F1.** (A) Representative confocal images of two recombined and mitoDsRed-transduced (red) adult-born neurons immunolabeled with doublecortin (DCX; white) in the DG of Ctrl (top) and *Nr2f1*-icKO (bottom) mice. Insets show magnification of mitoDsRed^+^ mitochondria in the reconstructed dendritic arbor (blue lines) of the two cells. (B,C) Total dendritic length (B; two-tailed unpaired Student's *t*-test, *P=0.0296) and the number of dendritic branches (C; Mann–Whitney *t*-test, *P=0.0153) in NR2F1-deficient mitoDsRed^+^DCX^+^YFP^+^ newborn neurons and Ctrl neurons; *n*=13 and *n*=14 cells, respectively, from three animals per genotype; at least three cells/animal were used for each analysis. (D) Linear density of dendritic mitochondria obtained by normalizing the total number of dendritic mitochondria to the total dendritic arborization length. Two-tailed unpaired Student's *t*-test, ***P=0.0001. (E) Relationship between the dendritic arborization length (*x*-axis) and the total number of dendritic mitochondria (*y*-axis) per mitoDsRed^+^DCX^+^YFP^+^ cell analyzed; linear regression with Pearson's correlation was performed with *R*^2^; *P*-values and best-fitted line equation are displayed. Note the shift in the regression fitted line of *Nr2f1*-icKO cells without changes in its steepness compared to that of the Ctrl fitted line. (F) Distribution of mitochondria throughout each branch of the dendritic arborization regardless of their branch order. Each dot represents a mitochondrion (*n*=379 mitochondria in Ctrl neurons; *n*=308 mitochondria in *Nr2f1*-icKO neurons). Mann–Whitney, *P*=0.3323. (G) Percentage of mitochondria belonging to the different branch order of the dendritic arborization in Ctrl and *Nr2f1*-icKO neurons. Mann–Whitney test: II, *P*=0.7000; III, *P*>0.9999; IV, *P*>0.9999; V, *P*=0.7000. (H) Representative confocal images showing high magnification of the mitoDsRed^+^ mitochondria in comparable dendritic branch levels of Ctrl and *Nr2f1*-icKO mitoDsRed^+^DCX^+^YFP^+^ adult-born neurons. (I) Cumulative frequency distribution for the length of dendritic mitochondria in control versus NR2F1-ablated mitoDsRed^+^DCX^+^YFP^+^ adult-born neurons. *n*=459 mitochondria from 13 control cells; *n*=279 mitochondria from 14 *Nr2f1*-icKO cells. Kolmogorov–Smirnov test, *P*<0.0001. (J) Quantification of dendritic mitochondrial morphologies according to four different subclasses based on their length (i.e. fragmented, <1 µm; elongated, from 1 to 2 µm; medium/large, from 2 to 3 µm; tubular, >3 µm). Mann–Whitney test: fragmented, ***P=0.001; elongated, *P*=0.6760; medium/large, *P*= 0.0917; tubular, ***P<0.0001. Each dot represents a cell. (K) Linear mitochondrial occupancy expressed as the percentage of dendritic length covered by the mitoDsRed staining. Mann–Whitney, ***P<0.0001. For all the analyses, *n*=13 (Ctrl) and *n*=14 (*Nr2f1*-icKO) cells from three animals per genotype and at least three cells/animal were used. Data are shown as mean±s.d. (B,C,D,G,J,K); best regression fitted line where the band represents the 95% confidence interval (E); box and whiskers plots with median (middle line), upper/lower quartiles, average (black crosses) and error bars ranging from minimum to maximum values (F). Each dot represents a cell (B-E,G,J,K) or a single mitochondrion (F). Scale bars: A, 20 µm; H,J, 5 µm.

To label mitochondria in adult-born hippocampal neurons, a retroviral vector encoding the mitochondria-targeted fluorescent protein DsRed (RV-mitoDsRed) was stereotaxically injected into the DG 2 weeks after induction by TAM. Mice were analyzed at 17 days post viral injection (Fig. 2A′), allowing recombined and transduced progenitors to develop into doublecortin-expressing neurons (i.e. DCX^+^mitoDsRed^+^YFP^+^ cells). Notably, whereas in Ctrl mice, YFP^+^ cells were virtually all NR2F1^+^, in *Nr2f1*-icKO mice, the population of YFP^+^ cells – including the specific subset transduced by the mitoDsRed virus – was substantially devoid of NR2F1 staining (Fig. 2B,C), thus validating *Nr2f1* inactivation in the aNSPC neuronal lineage of *Nr2f1*-icKO mice. Morphometric analyses were performed on DCX^+^mitoDsRed^+^YFP^+^ newborn neurons from *Nr2f1*-icKO and Ctrl mice showing comparable patterns of the dendritic tree (i.e. the primary dendrite branching in the outer part of the granule cell layer and the dendritic arborization spanning the molecular cell layer; Fig. 3A; Fig. S2A). We found no changes in the size of perikarya and primary dendrites between the two groups (Fig. S2B,C-C‴), but there was a modest difference in dendritic morphology. Specifically, the total dendritic length and the number of branching points were reduced in *Nr2f1*-icKO mice (Fig. 3B,C), indicating decreased complexity of the dendritic tree in NR2F1-deficient newborn neurons.

To initially assess the consequences of *Nr2f1* LOF on mitochondria in newborn DG neurons, we analyzed the mitochondrial occupancy expressed as the percentage of the area covered by the mitoDsRed^+^ signal over the area encompassing the perikaryon and the cone-shaped proximal portion of the primary dendrite (named ‘soma’ hereafter) (Fig. 2D,E; Fig. S2C,D). MitoDsRed^+^ mitochondria appeared densely packed and tightly organized into complex networks in the soma of Ctrl DCX^+^mitoDsRed^+^YFP^+^ newborn neurons (Fig. 2D; Fig. S2D). Interestingly, we observed a ∼20% reduction in the mitochondrial occupancy within the soma in NR2F1-depleted neurons compared to Ctrl ones (Fig. 2D,E). By exploiting the Mitochondrial Network Analysis (MiNA) toolset (Valente et al., 2017), we confirmed decreased mitochondrial mass (i.e. mitochondrial footprint), and we further revealed reduced complexity of the mitochondrial networks in NR2F1-deficient newborn neurons (Fig. 2G). Accordingly, we found a 28% reduction in the area covered by mitoDsRed+ mitochondria within the cylindrical portion of the primary dendrite of NR2F1-deficient newborn neurons (Fig. 2D,F; Fig. S2D). We next evaluated the mitochondrial content, morphology and distribution in the distal dendrites of DCX^+^mitoDsRed^+^YFP^+^ newborn neurons, where high-resolution confocal microscopy allowed the analysis of single mitoDsRed^+^ mitochondria (Fig. 3). We found a net reduction in the abundance of mitochondria in NR2F1-depleted neurons not only when quantified as the total number of mitochondria per dendritic arborization (Ctrl, 35.38±17.17; *Nr2f1*-icKO, 19.93±11.37; Mann–Whitney, *P*=0.0058), but also when normalized to the length of the dendritic arbor (Fig. 3D). Interestingly, we detected a linear correlation between the number of mitochondria and the dendritic arborization length in both Ctrl and *Nr2f1*-icKO neurons (Spearman correlation: Ctrl, *r*=0.7868, *P*=0.0021; *Nr2f1*-icKO, *r*=0.8326, *P*<0.001). However, the number of mitochondria was consistently lower in *Nr2f1*-icKO cells than in Ctrl cells of similar dendritic length (Fig. 3E). Overall, our analyses showed that the depletion of NR2F1 results in a global decrease in mitochondrial content in newborn hippocampal neurons. By comparing the different cell compartments, including the soma, primary dendrite and dendritic arbor, we found that the dendritic arbor was the most affected one in terms of mitochondria occupancy (Fig. S2G). Further analysis of the dendritic tree, by scoring each mitochondrion according to its position along the different dendritic segments, revealed no changes in their distribution between *Nr2f1*-icKO and Ctrl neurons (Fig. 3F,G; Fig. S2E). However, we found a reduction in the maximum length of single dendritic mitochondria in *Nr2f1*-icKO neurons compared to Ctrl ones (Fig. 3H,I). By classifying mitochondria into four different categories based on their length (adapted from Khacho et al., 2016), we detected an increase in fragmented mitochondria concomitantly with a reduction in tubular ones in the dendrites of *Nr2f1*-icKO neurons (Fig. 3J). Consistently, the estimation of the linear mitochondrial occupancy (i.e. the percentage of the dendritic length covered by the mitoDsRed^+^ signal) unveiled a marked decrease (∼55%) in NR2F1-depleted neurons compared to that in Ctrl neurons (Fig. 3K). Similar results were obtained by dividing the mitochondrial area by the dendritic length (Fig. S2F). To investigate whether NR2F1 loss triggered mitophagy, we performed colocalization analysis of the mitoDsRed staining with the lysosomal marker LAMP1 (Fig. S2H), revealing no differences between Ctrl and *Nr2f1*-icKO cells (Fig. S2I).

To support a direct role for NR2F1 in controlling mitochondrial morphology, we exploited a complementary genetic strategy to increase *Nr2f1* expression [i.e. gain of function (GOF)] by crossing the transgenic *CAGGS-lox-stop-lox-hCOUP-TFI* mouse line (Wu et al., 2010) to the *Glast::CreERT2* and *YFP* reporter line (herein *Nr2f1*-O/E; Fig. S3A) (Bonzano et al., 2018). Analysis of the mitochondrial content in the dendritic arborization of DCX^+^mitoDsRed^+^YFP^+^ newborn neurons in *Nr2f1*-O/E mice revealed the occurrence of longer mitochondria without changes in their number, leading ultimately to an overall increase in the linear dendritic mitochondrial occupancy in newborn granule neurons (Fig. S3B-D).

On the whole, data obtained by manipulating *Nr2f1* expression in newborn DG hippocampal neurons, together with GO annotations disclosing a stronger enrichment for a wide set of nuclear-encoded mitochondrial genes than that for other structural genes (e.g. cytoskeleton-related genes) as NR2F1 direct targets (Fig. 1), support a primary effect of NR2F1 on mitochondria in adult-born DG neurons.

### NR2F1 haploinsufficiency or conditional knockout lead to deregulation of key mitochondrial proteins

**Fig. 4.**
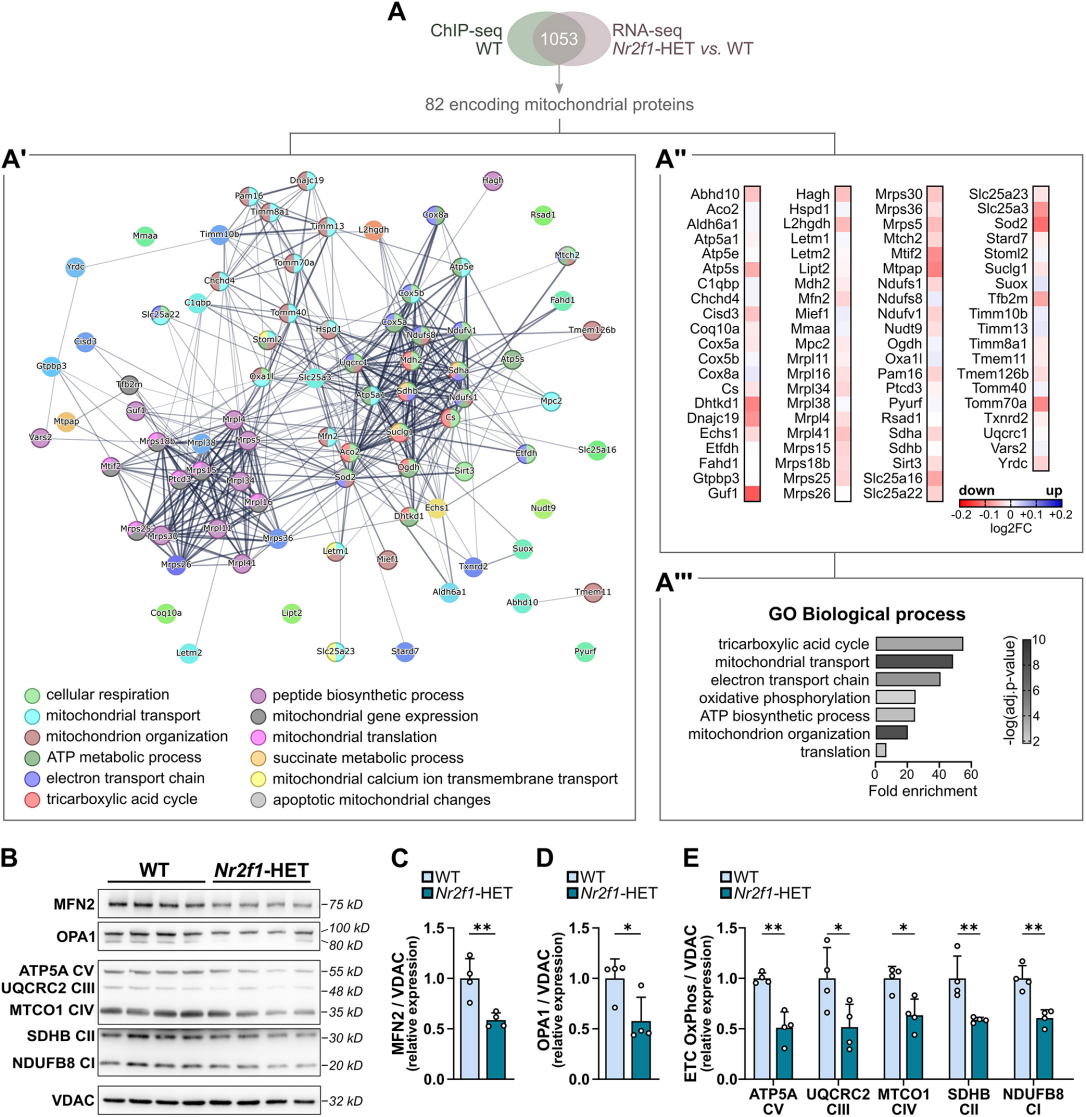
**NR2F1 haploinsufficiency induces changes in the expression of key mitochondrial proteins.** (A) Gene set enrichment analysis (GSEA) revealed that a large amount (82/1053) of nuclear-encoded mitochondrial proteins bound by NR2F1 shows altered expression in the hippocampi of adult constitutive heterozygous (*Nr2f1*-HET) mice. (A′) PPI graph of the 82 mitochondrial genes bound by NR2F1 and differentially expressed in *Nr2f1*-HET hippocampi. Nodes are colored according to the GO biological process annotation term. (A″) List of the mitochondrial genes belonging to the GSEA enriched core. Heatmap colors are based on the −log(fold change) of their expression in *Nr2f1*-HET versus WT hippocampi (dataset from Chen et al., 2020). (A‴) Bar graph illustrating the fold enrichment by annotation with GO term ranked for biological processes. Colors of the bars indicate the −log10(FDR) of the GO annotation term. (B) Representative western blots of isolated mitochondria proteins from whole-brain fresh tissue of WT and *Nr2f1*-HET mice, using antibodies against mitofusin-2 (MFN2), optic atrophy protein 1 (OPA1), subunits of each electron transport chain (ETC)/oxidative phosphorylation (OxPhos) complex, and voltage-dependent anion channel (VDAC). (C-E) Relative intensity levels of MFN2 (C), OPA1 (D) and ETC complexes [E; ATP synthase F1 subunit alpha (ATP5A); ubiquinol-cytochrome C reductase core protein 2 (UQCRC2); mitochondrially encoded cytochrome C oxidase I (MTCO1); succinate dehydrogenase complex iron sulfur Subunit B (SDHB); NADH:ubiquinone oxidoreductase subunit B8 (NDUFB8)] in WT and *Nr2f1*-HET isolated mitochondria. Data were normalized to VDAC and are shown relative to WT samples. Error bars indicate mean±s.d. Each dot represents an animal. *n*=4 WT/*Nr2f1*-HET mice. Two-tailed unpaired Student's *t*-test, *P<0.05, **P<0.01.

To further investigate whether loss of NR2F1 recruitment to its target genes affect their expression, which in turn could lead to the observed mitochondrial phenotype, we ran a gene set enrichment analysis (GSEA) by integrating our ChIP-seq dataset with previously published transcriptome data on the hippocampi of adult mice constitutively heterozygous for NR2F1 (*Nr2f1*-HET) (Chen et al., 2020), a validated BBSOAS mouse model (Bertacchi et al., 2019a, 2020; Jurkute et al., 2021; Tocco et al., 2021). We considered NR2F1 targets as a ‘gene set’ and verified that those genes were predominantly downregulated [normalized enrichment score, <−2.9; false discovery rate (FDR), <0.001] (Fig. S4A). Among the 1053 genes in the GSEA core enrichment set, ∼8% (82/1053) code for mitochondrial proteins (Fig. 4A) that form highly interacting networks to ensure proper mitochondrial homeostasis and function (Fig. 4A′). Remarkably, the expression of transcripts for these mitochondrial proteins was regulated in the *Nr2f1*-HET hippocampi, where 70% and 30% showed downregulation and upregulation, respectively (Fig. 4A″). GO term annotation of these genes further corroborated a pivotal and direct role for NR2F1 in fine tuning the expression of nuclear-encoded mitochondrial proteins. Indeed, strong enrichment (fold enrichment, >7) was observed in factors controlling a plethora of biological processes needed for proper mitochondrial functioning, such as (1) the organization of mitochondria (i.e. assembly/arrangement/disassembly of a mitochondrion, mitochondrial morphogenesis, dynamics and their distribution); (2) mitochondrial transport (i.e. the transport of substances into, out of or within a mitochondrion, as well as the targeting of protein into mitochondria; (3) mitochondria-related metabolism and cellular respiration [i.e. tricarboxylic acid (TCA) cycle, electron transport chain (ETC)/OxPhos]; and (4) synthesis of new mitochondrial components (Fig. 4A‴).

In order to directly assess changes in mitochondrial proteins due to NR2F1 haploinsufficiency, we isolated mitochondria from the whole brain of adult *Nr2f1*-HET and WT Ctrl mice, and we quantified the levels of selected key mitochondrial proteins by western blot (WB) assay (Fig. 4B-E). Based on the mitochondrial phenotype observed in *Nr2f1*-icKO neurons, we initially evaluated the levels of the mitochondrial membrane proteins MFN2 and OPA1, which are needed for mitochondrial fusion (Quintana-Cabrera and Scorrano, 2023). We found that both MFN2 and OPA1 protein levels normalized to mitochondrial porin [i.e. the voltage-dependent anion channel (VDAC)] were markedly reduced in *Nr2f1*-HET mitochondrial extracts compared to those in Ctrl mitochondrial extracts (Fig. 4B,C,D). Based on the enrichment of genes involved in cellular respiration among the GSEA core enrichment set (Fig. 4A‴), we next wondered whether brain mitochondria of NR2F1 haploinsufficient mice showed changes in ETC and OxPhos components necessary for cellular respiration and oxidative metabolism. We revealed that protein levels of all analyzed ETC and OxPhos components, normalized to VDAC, were reduced in *Nr2f1*-HET mitochondrial extracts (Fig. 4B,E).

**Fig. 5.**
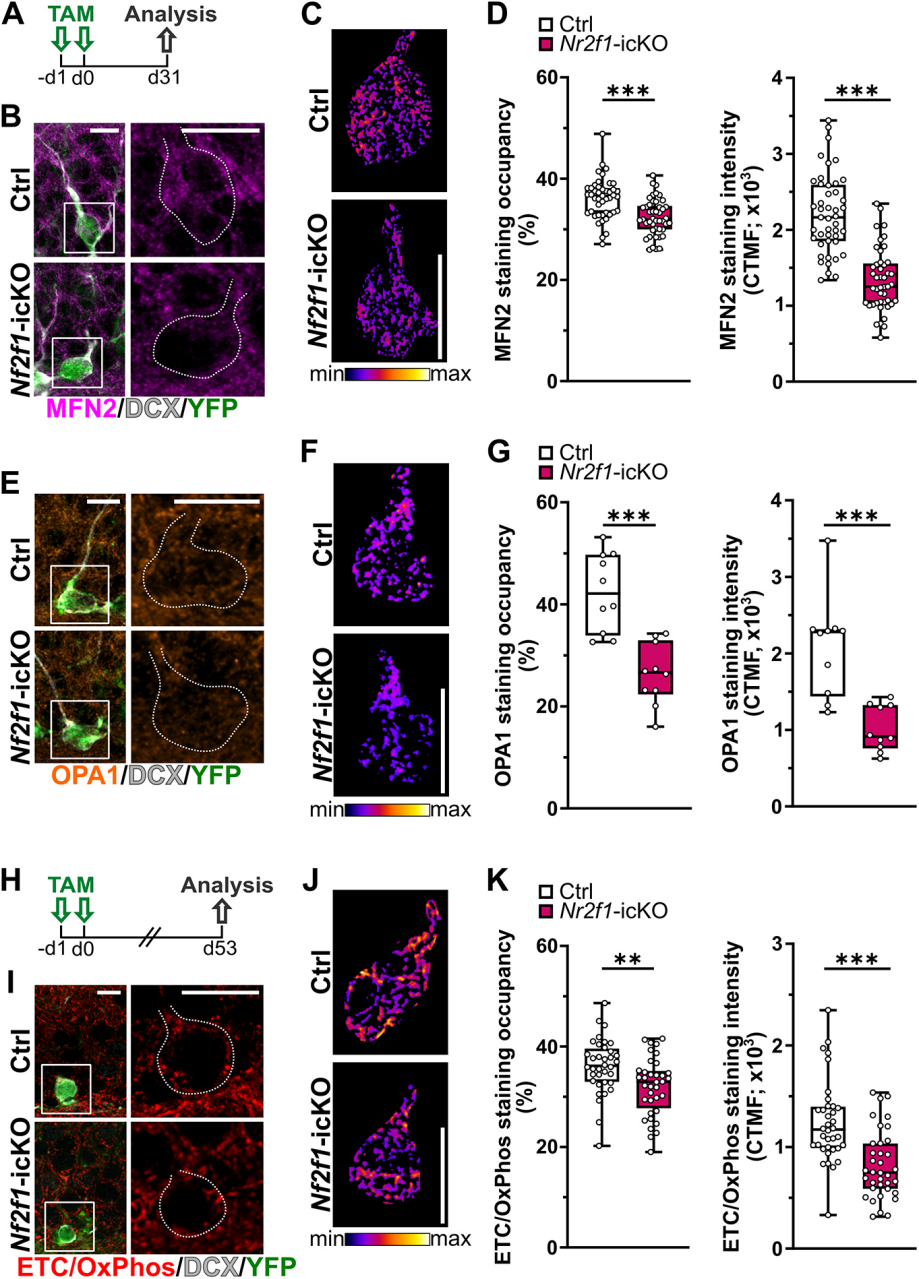
**NR2F1 conditional knockout leads to the deregulation of key mitochondrial proteins in a cell-autonomous manner.** (A) Experimental design. (B) Representative confocal images of the DG immunostained for YFP (green), MFN2 (magenta) and DCX (white) in Ctrl (top) and *Nr2f1*-icKO (bottom) mice 1 month after TAM administration. (C) Representative heatmap images of MFN2^+^ signal in control (top) and NR2F1-deficient (bottom) recombined newborn neurons. (D) Left: mitochondrial MFN2 occupancy expressed as the percentage of the soma area covered by the MFN2^+^ signal. Mann–Whitney test, ***P<0.0001. Right: mitochondrial MFN2 staining intensity normalized to the area occupied by MFN2 staining in the soma [expressed as cell threshold mitochondrial fluorescence (CTMF)]. Two-tailed unpaired Student's *t*-test, ***P<0.0001. *n*=45 cells from three Ctrl mice; *n*=45 cells from three *Nr2f1*-icKO mice. (E) Representative confocal images of the DG immunostained for YFP (green), OPA1 (orange) and DCX (white) in Ctrl (top) and *Nr2f1*-icKO (bottom) mice 1 month after TAM administration. (F) Representative heatmap images of OPA1^+^ signal in control (top) and NR2F1-deficient (bottom) recombined newborn neurons. (G) Left: mitochondrial OPA1 occupancy expressed as the percentage of the soma area covered by the OPA1^+^ signal. Mann–Whitney test, ***P=0.0002. Right: mitochondrial OPA1 staining intensity normalized to the area occupied by OPA1 staining in the soma (expressed as CTMF). Two-tailed unpaired Student's *t*-test, ***P=0.0005. *n*=10 cells from three Ctrl mice; *n*=10 cells from three *Nr2f1*-icKO mice. (H) Experimental design. (I) Representative confocal images of the DG immunostained for YFP (green), ETC/OxPhos (red) and DCX (white) in Ctrl (top) and *Nr2f1*-icKO (bottom) mice 7.5 weeks after TAM administration. (J) Representative heatmap images of ETC/OxPhos^+^ signal in control (top) and NR2F1-deficient (bottom) recombined newborn neurons. (K) Left: mitochondrial ETC/OxPhos occupancy expressed as the percentage of the soma area covered by the ETC/OxPhos^+^ signal. Mann–Whitney test, **P=0.0023. Right: mitochondrial ETC/OxPhos staining intensity normalized to the area occupied by ETC/OxPhos staining in the soma (expressed as CTMF). Mann–Whitney test, ***P<0.0001. *n*=36 cells from three Ctrl mice; *n*=10 cells from three *Nr2f1*-icKO mice. The dotted lines in B, E and I (right) delimit the cell bodies of DCX^+^YFP^+^ neurons shown at lower magnification in the white boxes on the left. Data are shown as box and whiskers plots with median (middle line), upper and lower quartiles, and error bars ranging from minimum to maximum values. Each dot represents a cell. Scale bars: 10 μm.

Thus, the observed changes in mitochondrial protein composition are in line with the transcriptomic data indicating impairment of mitochondrial fusion and cellular respiration in NR2F1 haploinsufficient mice. To confirm that downregulation of those mitochondrial factors occurs due to cell-intrinsic mechanisms, we used the *Nr2f1*-icKO model to perform a densitometric analysis of immunofluorescence staining for MFN2, OPA1 and ETC/OxPhos within the soma of DCX^+^YFP^+^ newborn neurons lacking NR2F1 in comparison to the soma of Ctrl ones (Fig. 5). For this assay, we exploited a custom-made workflow, allowing analysis of mitochondrial staining coverage and intensity within the soma+shaft compartment of newborn neurons (see Materials and Methods). First, assessment of both MFN2 and OPA1 fluorescence labeling was carried out 1 month after TAM administration in *Nr2f1*-icKO mice (Fig. 5A). In line with the observed reduction in mitochondrial occupancy in *Nr2f1*-icKO newborn neurons (Fig. 2E), we found a reduction in the percentage of area occupied by both the MFN2 and OPA1 mitochondrial signals compared to that in Ctrl newborn neurons (Fig. 5D,G). Notably, analysis of the intensity of mitochondrial staining normalized to the area occupied by the signals [cell threshold mitochondrial fluorescence (CTMF)] revealed a marked decrease in MFN2 and OPA1 (∼40% and 50% decrease, respectively) in *Nr2f1*-icKO mice (Fig. 5C,D,F,G). Next, for the analysis of ETC/OxPhos components, adult *Nr2f1*-icKO and Ctrl brain sections were collected 7.5 weeks after TAM administration (Fig. 5H) and immunolabeled with the same ETC/OxPhos antibody mix used for WB assays. Interestingly, *Nr2f1*-icKO newborn granule neurons showed a net reduction in ETC/OxPhos staining occupancy as well as a decrease in ETC/OxPhos staining intensity when normalized to the ETC/OxPhos^+^ mitochondrial area (CTMF, ∼45% reduction) (Fig. 5I,J,K). Taken together, these data further corroborate our hypothesis that NR2F1 shapes the mitochondrial proteome in mouse neurons in a cell-autonomous manner.

### NR2F1 loss in the adult DG neurogenic niche impairs functional integration and long-term survival of adult-born granule neurons

Mitochondria control and integrate several cellular processes, including proliferation, differentiation, migration and survival. Specifically, in the adult neurogenic DG, defective mitochondria in newborn neurons have been associated with dendritic and synaptic impairments, which eventually lead to behavioral deficits in learning and memory performances (Khacho et al., 2016).

**Fig. 6.**
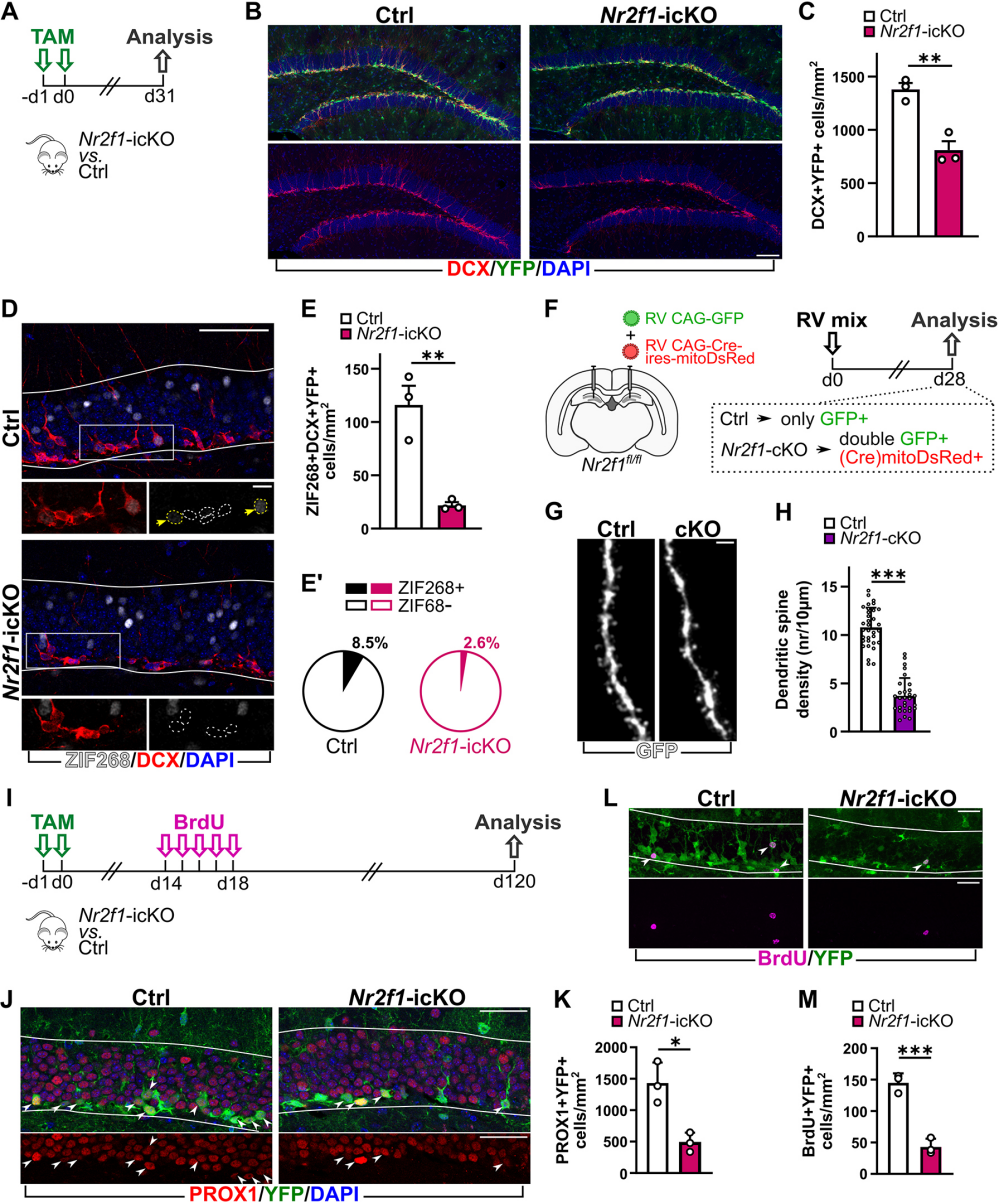
**NR2F1 loss in the adult DG neurogenic niche impairs functional integration and long-term survival of adult-born granule neurons.** (A) Experimental design. (B) Representative confocal images of the DG immunostained for YFP (green) and DCX (red), and counterstained with DAPI (blue), in Ctrl (top) and *Nr2f1*-icKO (bottom) mice 1 month after TAM administration. (C) Density of DCX^+^YFP^+^ neurons in the DG of Ctrl and *Nr2f1*-icKO mice. Two-tailed unpaired Student's *t*-test, **P=0.0051. (D) Representative confocal images of the DG immunostained for ZIF268 (EGR1) (white) and DCX (red), and counterstained with DAPI (blue), in Ctrl (top) and *Nr2f1*-icKO (bottom) mice 1 month after TAM administration. Micrograph at high magnification shows DCX^+^ cells negative (white dashed line contour) or positive (yellow dashed line contour and arrows) for ZIF268. (E,E′) Numbers of DCX^+^YFP^+^ neurons expressing ZIF268. These data are expressed as density of ZIF268^+^DCX^+^YFP^+^ neurons (E; two-tailed unpaired Student's *t*-test, **P=0.0062) and as percentage among the whole DCX^+^YFP^+^ cellular population (E′; Mann–Whitney test, *P*=0.1000) in the DG of Ctrl and *Nr2f1*-icKO mice. (F) Experimental design. (G) Representative high-resolution confocal images of the GFP^+^ dendritic spines in comparable levels of dendrites in Ctrl and *Nr2f1*-icKO 28-day-old DG neurons. (H) Density of dendritic spines in Ctrl and *Nr2f1*-icKO GFP*^+^* dendrites. Mann–Whitney test, ***P<0.0001. *n*=33 dendritic segments from five GFP^+^ Ctrl cells; *n*=29 dendritic segments from five mitoDsRed^+^GFP^+^ *Nr2f1*-icKO cells. (I) Experimental design. (J) Representative confocal images of the DG immunostained for YFP (green) and PROX1 (red), and counterstained with DAPI (blue), in Ctrl (left) and *Nr2f1*-icKO (right) mice 4 months after TAM administration. (K) Density of PROX1^+^YFP^+^ neurons in the DG of Ctrl and *Nr2f1*-icKO mice. Two-tailed unpaired Student's *t*-test, *P=0.0265. (L) Representative confocal images of the DG immunostained for YFP (green) and BrdU (magenta) in Ctrl (top) and *Nr2f1*-icKO (bottom) mice 4 months after TAM administration. (M) Density of BrdU^+^YFP^+^ cells in the DG of Ctrl and *Nr2f1*-icKO mice. Two-tailed unpaired Student's *t*-test, ***P=0.0008. Solid white lines in D, J and L define the area of the DG GCL and SGZ used for the quantification of cell densities. White arrowheads in J and L indicate double-labeled cells. Data are shown as mean±s.d. Each dot represents an animal (C,E,K,M) or a dendritic segment (H). Scale bars: B, D (low magnification), 50 µm; D (high magnification) 10 µm; G, 2 µm; J, 50 µm, L, 30 µm.

We thus wondered whether the observed mitochondrial defects caused by *Nr2f1* LOF in DG newborn neurons were coupled to dysfunctional consequences at the cellular level. To evaluate whether NR2F1 deficiency in adult-born DG neurons might impact on their functional integration in the hippocampal circuits that is fundamental for their proper maturation and survival (Gonçalves et al., 2016), we analyzed *Nr2f1*-icKO mice 1 month after Cre-recombinase induction by TAM (Fig. 6A). In these mice, the number of DCX^+^YFP^+^ neurons in the DG was strongly reduced compared to that in Ctrl mice (Fig. 6B,C), indicative of impaired neurogenesis, which is consistent with previous findings in *Nr2f1*-icKO mice at shorter chase times after TAM administration (Bonzano et al., 2018).

Notably, quantification of expression of the immediate early gene *Zif268* (*Egr1*), a proxy for neural activity (Veyrac et al., 2013), in DCX^+^ newborn neurons revealed a net decrease in the fraction of double-positive ZIF268^+^DCX^+^ cells in *Nr2f1*-icKO mice compared to that in Ctrl mice (80% decrease; Fig. 6D-E′), indicating defective cellular activation that could be caused by altered synaptic integration in hippocampal circuitry. To directly assess this possibility, we stereotaxically injected a mixture of retroviral vectors in the DG of *Nr2f1*-floxed mice to label the whole cytoplasm (including dendritic spines) by GFP in a cohort of newborn neurons and to concomitantly induce Cre-driven NR2F1 loss in a subset of them (double-labeled GFP and mitoDsRed cells; *Nr2f1*-icKO cells) (Fig. 6F). Animals were then euthanized 4 weeks after viral transduction, i.e. a time point at which labeled neurons exhibit elaborated dendritic trees typical of mature granule neurons (Zhao et al., 2006) and show dendritic protrusions forming synapses, to analyze the abundance of dendritic spines as an indicator of their functional connectivity. Remarkably, we found a dramatic reduction in the density of dendritic spines in NR2F1-depleted neurons (Fig. 6G,H). This finding, together with decreased expression of the immediate early gene *Zif268*, indicates that NR2F1 loss in adult-born neurons cell-autonomously affects the neurons’ ability to functionally integrate into the pre-existing hippocampal circuitry.

Because the survival of adult-born neurons depends on their synaptic integration (Tashiro et al., 2007), we examined whether NR2F1 inactivation was associated with alterations in the long-term survival of adult-born neurons. Adult Ctrl and *Nr2f1*-icKO mice in the Glast lineage were injected with 5-bromo-2-deoxyuridine (BrdU) 2 weeks after TAM administration, and the number of recombined mature neurons as well as the amount of BrdU^+^ surviving cells were analyzed 4 months after the beginning of the protocol (Fig. 6I). Interestingly, the significant reduction in the number of PROX1^+^YFP^+^ mature granule neurons (60% decrease; Fig. 6J,K) was mirrored by a decrease in the density of BrdU^+^YFP^+^ cells (Fig. 6L,M) within the DG of *Nr2f1*-icKO mice compared to that in Ctrl mice, indicating defective long-term survival of NR2F1-deficient neurons.

## DISCUSSION

BBSOAS is a neurodevelopmental disorder caused by mutations in the *NR2F1* gene, associated with several symptoms, including optic atrophy, hypotonia, seizure and ID, that might be compatible with mitochondrial dysfunction in the nervous system. Interestingly, a possible implication of mitochondrial dysfunction in the BBSOAS was proposed by two independent clinical investigations reporting defective function of the ETC/OxPhos machinery in the muscles of two BBSOAS patients (Hobbs et al., 2020; Martín-Hernández et al., 2018). However, whether the mitochondrial involvement is a general mechanism in BBSOAS and how *NR2F1* haploinsufficiency might lead to mitochondria-related functional defects in neurons are completely unknown.

It is well known that mitochondria have essential roles in both bioenergetic and non-energetic biological processes and are increasingly recognized as major players in neuronal development and function, including neural stem cell fate, dendritogenesis, neuronal maturation, synaptic transmission and plasticity (Faits et al., 2016; Iwata et al., 2023; Khacho et al., 2016; 2019; Kimura and Murakami, 2014; Rangaraju et al., 2019a, b; Steib et al., 2014). Accordingly, mitochondrial dysfunction dramatically contributes to the pathogenesis of various neurodegenerative and neurodevelopmental disorders (Jurcau, 2021; Monzio Compagnoni et al., 2020; Rojas-Charry et al., 2021). In mammals, the mitochondrial proteome comprises more than 1100 proteins that are predominantly encoded by the nuclear genome, translated in the cytosol and imported into mitochondria, whereas few components (∼1%; e.g. MTCO1) are encoded by the mitochondrial DNA (Pfanner et al., 2019; Rath et al., 2021). Thus, mitochondrial biogenesis and function requires the coordinated action of transcription factors regulating a large number of mitochondrial genes in the nucleus. In this study, by genome-wide and *in silico* analyses, we provide strong evidence supporting direct involvement of NR2F1 in this mitochondrial gene expression regulatory network in the adult mouse brain. First, ChIP-seq data showed that NR2F1 binds to promoters predominantly in a chromatin-permissive state, in line with a previous study reporting high association of NR2F1/2 with open chromatin characterized by high levels of p300 and H3K27ac in neural crest cells (Rada-Iglesias et al., 2012). Interestingly, mitochondrial factors emerged as the most enriched among the NR2F1 nuclear genomic targets. Moreover, NR2F1 mitochondrial putative targets form a highly interconnected network of functional and physical protein associations, as revealed by STRING analysis, suggesting that NR2F1 may contribute to a coordinated regulation of multiple aspects of mitochondrial biogenesis and function. Accordingly, a GSEA analysis conducted by interpolating our ChIP-seq dataset with previously published transcriptome data on adult *Nr2f1*-HET mice (Chen et al., 2020) shows that, among the direct NR2F1 putative genomic targets, many are downregulated upon NR2F1 haploinsufficiency, of which 82 encode mitochondrial proteins. Interestingly, those proteins control key biological processes, ranging from mitochondrial morphogenesis, dynamics and transport to metabolism and cellular respiration. Previously published microarray data on *Nr2f1*-null embryonic neocortex (Montemayor et al., 2010) show altered expression of nuclear genes encoding mitochondrial proteins (e.g. *Slc25a1*, *Echs1*, *Mrpl45*, *Timm23*), thus strengthening our findings.

We have previously demonstrated that NR2F1 conditional deletion in aNSPCs by the Cre-loxP system leads to defective neurogenesis in the hippocampal DG (Bonzano et al., 2018). This occurs without alteration in neural stem cell/progenitor proliferation rate or short-term newborn cell survival within 2 weeks after the proliferative event (Bonzano et al., 2018). Here, by exploiting the same system associated with retroviral-mediated labeling of mitochondria in adult-born DG neurons (Steib et al., 2014), we provide strong evidence of a significant mitochondrial phenotype due to *Nr2f1* LOF. Specifically, our morphometric analyses clearly showed a reduction in the mitochondrial mass as well as in the complexity of the mitochondrial networks in the soma of NR2F1-depleted neurons. In addition, in the dendritic tree of those neurons, we observed an even stronger defect in mitochondrial occupancy accompanied by increased fragmented mitochondria. However, no alteration in mitochondrial distribution was detected in this cell compartment. Interestingly, NR2F1 overexpression in DG newborn neurons showed an opposite mitochondrial phenotype, leading to reduced fragmentation and to an overall increase in the dendritic mitochondrial mass in newborn granule neurons. Mitochondria undergo continuous morphological transition by dynamic processes thanks to the coordinated action of proteins promoting mitochondrial fission [e.g. Drp1 (DNM1L), FIS1] and fusion (e.g. MFN1/2, OPA1), as well as their transport [e.g. Miro (RHOT), Milton) (Course and Wang, 2016; Giacomello et al., 2020; Misgeld and Schwarz, 2017). Based on the mitochondrial phenotype we uncovered – characterized by increased mitochondrial fragmentation but no changes in positioning – we conducted further analyses on factors involved in mitochondrial fusion. Indeed, it is well known that altered expression and function of those factors leads to permanent changes in the overall shape of mitochondria (Chan, 2020), and MFN2 downregulation was reported to induce mitochondrial fragmentation and decreased mitochondrial mass in differentiated excitatory neurons *in vitro* (Fang et al., 2016). Biochemical quantification of mitochondrial MFN2 and OPA1 protein levels revealed that both were downregulated in the brain of *Nr2f1*-HET mice. Moreover, *in situ* analysis of immunofluorescence staining for MFN2 and OPA1 in NR2F1-depleted newborn neurons showed decreased area covered and intensity for both proteins. Notably, we found that NR2F1 binds to a promoter region close to the TSS of the *Mfn2* gene, which is associated with the H3K4me3 in the adult neural tissue (Fig. S4B), strongly supporting a direct and positive regulation of *Mfn2* expression by NR2F1. On the other hand, *Opa1* did not emerge among the direct NR2F1 putative targets. However, because of the extensive network of nuclear-encoded mitochondrial factors that could be potentially controlled by NR2F1, *Opa1* downregulation may be the result of indirect mechanisms regulating the expression and protein stability of OPA1 and/or its import into the inner mitochondrial membrane. Alteration in OPA1 expression in BBSOAS models is particularly interesting considering that *OPA1* haploinsufficiency is the major genetic pathological mechanism of autosomal-dominant optic atrophy in humans (Lenaers et al., 2021) and that optic atrophy in BBSAOS patients is among the most common clinical features. In light of our findings, the mitochondrial abnormalities recently observed in the optic nerve and retina of constitutive *Nr2f1*-knockout/*Nr2fl*-HET mice (Bertacchi et al., 2019a) might be a direct consequence of NR2F1 altered function that could contribute to the optic atrophy observed in BBSOAS mouse models.

The brain mitochondria in *Nr2f1* haploinsufficient mice as well as in *Nr2f1*-depleted newborn neurons were also found to be defective in ETC/OxPhos components. Notably, mitochondria complexes I-V were all found decreased by WB analysis in the brain of *Nr2f1*-HET mice. This wide effect of NR2F1 depletion on ETC/OxPhos components is noteworthy, because only three of them (i.e. NDUFB8 of complex I, SDHB of complex II and ATP5A of complex V) are direct NR2F1 genomic targets, whereas the mitochondrial DNA-encoded MTCO1 (complex IV) and the nucleus-encoded UQCRC2 (complex II) are not among the direct NR2F1 targets based on our ChIP-seq. This implicates indirect mechanisms, as suggested for OPA1, involving other factors that, in turn, may control their expression. Interestingly, some key components of the core transcriptional network, including DNA-binding factors that target mitochondrial genes in the nucleus, are direct genomic targets for NR2F1. Among them, nuclear respiratory factor 1 (NRF1), which is one of the major activators for the expression of key metabolic nuclear genes required for respiration, as well as mitochondrial DNA transcription and replication, and estrogen-related receptor alpha (ERRα), which regulates an array of nuclear genes devoted to mitochondrial functions and also numerous mitochondrial DNA genes (Hock and Kralli, 2009). An additional indirect mechanism especially for the observed MTCO1 downregulation might involve the transcriptional control of genes crucial for mitochondrial DNA translation and mitochondrial transduction (e.g. the mitochondrial ribosomal proteins).

Furthermore, mitochondria dynamics and function are tightly linked to the development and maintenance of dendritic architecture, synaptic integration and neuronal survival (Li et al., 2004; Dickey and Strack, 2011; Faits et al., 2016; López-Doménech et al., 2016; Divakaruni et al., 2018; Steib et al., 2014; Kimura and Murakami, 2014; Han et al., 2020; Iwata et al., 2023). Thus, the observed mitochondrial phenotype in newborn neurons lacking NR2F1 might underlie the differences we observed in terms of dendritic length and the overall complexity of the dendritic arbor. Interestingly, these effects are reminiscent of the consequences previously reported following MFN2 manipulations on excitatory neurons both *in vitro* and *in vivo* (Faits et al., 2016; Kimura and Murakami, 2014). Going beyond the role of mitochondrial dynamics in neurite outgrowth, Iwata et al. (2023) have recently shown that interfering with mitochondrial metabolism in mouse neurons, either by pharmacological inhibition of the TCA cycle or ETC complex I activity, leads to a decrease in dendritic length and neuronal complexity *in vitro.* Moreover, a previous study demonstrated that hampering mitochondrial dynamics and homeostasis dramatically affects the morphogenesis of spines *in vitro* (Li et al., 2004). The importance of dendritic mitochondria in the morphogenesis and plasticity of spines and synapses is further corroborated by the demonstration that mitochondria stabilized near synapses serve as local energy supplies to fuel synaptic plasticity in hippocampal neurons *in vitro* (Rangaraju et al., 2019a; Bapat et al., 2023 preprint) and that dendritic spine plasticity is intimately correlated with the remodeling of the proximal mitochondrial network in pyramidal neurons *in vivo* (Divakaruni et al., 2018).

Thus, our findings showing a marked reduction in the number of dendritic spines associated with reduced expression of the immediate early gene *Zif268* in NR2F1-depleted neurons might be the direct consequence of dysfunctional mitochondria that, in the long term, would also lead to impaired survival of NR2F1-depleted neurons, as we observed in our study. Accordingly, the observed downregulation of the ETC/OxPhos machinery, as well as defective survival of adult-born neurons months after their generation, recapitulate a condition recently discovered in adult neurons lacking MFN2, in which a mitochondrial phenotype and neurodegeneration occur months after MFN2 loss (Han et al., 2020).

Overall, our data point to mitochondrial dysfunction in neural tissue as a potential key pathological mechanism in BBSOAS, suggesting that the current estimation of mitochondrial involvement in BBSOAS patients might be underestimated. As the homology between human and mouse NR2F1 is very high – especially in the DNA-binding domain, with 100% amino acid sequence homology (Bertacchi et al., 2019b; Qiu et al., 1995) – their functions and targets are likely to be conserved in both species, strongly supporting a major role of mitochondrial dysfunction in the BBSOAS neuropathology. Altered NR2F1 expression has also been reported in Down syndrome human-derived neural cells (Bhattacharyya et al., 2009; Halevy et al., 2016) as well as in neurodegenerative disorders, including in mouse models of Alzheimer's and Parkinson's diseases (Walter et al., 2021; Zheng et al., 2020). Therefore, our findings open new perspectives for future investigation on the role of NR2F1 in mitochondrial dysfunction associated with the pathogenesis and progression of neurological disorders.

## MATERIALS AND METHODS

### Animals and treatments

*In vivo* experiments were performed on 2- to 3-month-old C57BL/6J mice (Charles Rivers Laboratories) of both sexes. Triple transgenic mice (C57BL/6J background) were also used to manipulate NR2F1 expression through TAM administration. *Glast::CreERT2^+/wt^;R26-loxP-stop-loxP-YFP^yfp+/yfp+^;Nr2f1^fl/fl^* (*Nr2f1*-icKO) mice were used for *in vivo* LOF experiments, and *Glast::CreERT2^+/wt^;R26-loxP-stop-loxP-YFP^yfp+/yfp+^;CAG-S-loxP-stop-loxP-hCOUP-TFI^+/wt^* (*Nr2f1*-O/E) mice were used for analyses of *in vivo* NR2F1 overexpression (see Bonzano et al., 2018). *Glast::CreERT2^+/wt^;R26-loxP-stop-loxP-YFP^yfp+/yfp+^* mice were used as Ctrl mice. For activation of the *CreERT2*-recombinase in the Glast^+^ lineage, animals were administered TAM (T-5648, Sigma-Aldrich) at a dose of 2.5 mg/mouse/day dissolved into corn-oil (Sigma-Aldrich) by means of intraperitoneal injections for 2 consecutive days at the age of 2 months (Bonzano et al., 2018). Adult *Nr2f1^fl/fl^* mice were used for cell-autonomous LOF experiments obtained by RV-Cre-mitoDsRed stereotaxic injections within the adult DG. Adult (8-month-old) constitutive *Nr2f1* heterozygous mice (i.e. *Nr2f1^wt/null^*, named *Nr2f1*-HET) were generated and genotyped as previously described (Jurkute et al., 2021). Littermates of *Nr2f1*-HET mice with WT *Nr2f1* alleles were used as Ctrl mice [i.e. *Nr2f1^wt/wt^* (WT)]. Both *Nr2f1*-HET and WT littermates were bred in a 129S2/SvPas background.

Mice were housed under standard laboratory conditions (*n*=2-4 mice/standard cage) with basic recommended environmental enrichment (paper tubes and litter, igloo) under a 12 h light/dark cycle with access to food and water *ad libitum*. All procedures were conducted in accordance with the Guide for the Care and Use of Laboratory Animals of the European Community Council Directives (2010/63/EU and 86/609/EEC) and approved by local bioethics committees, the Italian Ministry of Health (Authorization number 864/2018-PR, protocol E6B5E.3; Authorization number 49/2023-PR, protocol E6B5E.8) and the French Ministry of Education, Research and Innovation CIEPAL NCE/2019–548 (Nice) under authorization #15 349 and #15 350.

BrdU (Sigma-Aldrich) was intraperitoneally injected (100 mg/kg BrdU per injection, calculated based on the weight of the animal) after TAM treatments in the Glast-dependent lineage. For analysis of long-term cell survival, five BrdU injections, 24 h apart, were performed on day 14-18 after the last TAM treatment on animals that received 2 days TAM administration; animals were euthanized 102 days after the last BrdU injection.

### Retroviral production

Replication-deficient recombinant murine Moloney leukemia (MML) retroviruses specifically transduce proliferating cells and allow the dating of their birth, as well as label precursor cells and their progeny in the adult hippocampal neurogenic lineage (Zhao et al., 2006). pCAG-GFP has previously been described (Jagasia et al., 2009). pCAG-IRES-mitochondrial Discosoma Red (mitoDsRed) was generated from the pCAG IRES-GFP vector (Jagasia et al., 2009) by replacing the GFP coding sequence with cDNA for mitochondrially targeted DsRed. pCAG-Cre-IRES-mitoDsRed was generated from the pCAG-Cre-IRES-GFP vector by replacing the GFP coding sequence with cDNA for the mitoDsRed. Retroviruses were generated as described previously (Steib et al., 2014; Zhao et al., 2006). Virus-containing supernatant was harvested four times every 48 h after transfection and concentrated by two rounds of ultracentrifugation. Viral titers were determined by transduction of HEK293T cells for 72 h with a limiting dilution of MMLV suspension and counting of reporter expressing cell spots under a fluorescent microscope (Leica Microsystems). Titers applied were ∼5×10^8^ colony-forming units (CFU)/ml (Zhao et al., 2006).

### Surgical procedure for retroviral injection

Two weeks after TAM administration, adult mice were anesthetized in a constant flow of isoflurane (3%) in oxygen, positioned in a stereotaxic apparatus (Stoelting) and injected with a pneumatic pressure injection apparatus (Picospritzer II, General Valve Corporation). The skull was exposed by an incision in the scalp, and a small hole (∼1 mm) was drilled through the skull. Then, 0.8 µl retrovirus-mitoDsRed was injected in the DG using a sharpened glass capillary at the following stereotaxic coordinates: −2 mm (antero-posterior), 1.5 mm (lateral) to Bregma and −2.0 mm below the surface of the skull. Mice (*n*=3/4 genotype/experiments) were analyzed 17 days after retroviral injection. For double injections with pCAG-GFP and pCAG-Cre-IRES-mitoDsRed into the DG of *Nr2f1^fl/fl^* mice, a virus particle suspension (total volume, 1 µl) of a mixture of the two viruses (1:1) with a concentration of ∼1×10^8^ CFU/μl each was used, and mice were analyzed 28 days after retroviral injection.

### Tissue preparation and immunofluorescence staining

For immunostaining, adult mice were deeply anesthetized with an intraperitoneal injection of a mixture of tiletamine and zolazepam (40-80 mg/kg; Zoletil, Virbac) and perfused transcardially with ice-cold 0.9% saline solution followed by ice-cold 4% paraformaldehyde (PFA) in 0.1 M phosphate buffer (PB), pH 7.4. Brains were removed from the skull, post-fixed for 4 h in the same PFA solution, cryoprotected in a 30% sucrose solution (in 0.1 M PB, pH 7.4), embedded with cryo-embedding matrix (OCT; Killik, BioOptica), frozen at −80°C and finally sectioned using a cryostat (Leica Microsystems). Free-floating coronal serial sections (50 µm thick for the hemisphere ipsilateral to the viral injection; 40 µm thick for the contralateral hemisphere) were collected in series on multi-well dishes (six to eight wells per animal). Sections were stored at −20°C in antifreeze solution (30% ethylene glycol, 30% glycerol, 10% PB 0.2 M, pH 7.4) until use. Immunofluorescence reactions were performed on free-floating coronal serial sections as detailed below: sections were incubated in blocking solution [0.01 M PBS, pH 7.4, 1% or 2% Triton X-100 for 40 µm- and 50 µm-thick slices, respectively, and 10% normal serum of the same species of the secondary antibody, i.e. normal donkey serum (NDS)] for 2 h at room temperature. Afterwards, slices were incubated for 48 h at 4°C with primary antibodies (see Table S2) diluted in 0.01 M PBS (pH 7.4), 1-2% Triton X-100 (for 40 µm- and 50 µm-thick slices, respectively) and 1% NDS. For immunostaining of MFN2, sections were subjected to antigen retrieval. Slices were incubated in 10 mM Tris, 1 mM EDTA, 0.05% Tween 20 for 2 min at 99°C and washed three times with MilliQ water, followed by three washing steps with 0.01 M PBS (pH 7.4) prior to incubation with primary antibody. Sections were washed in PBS and incubated for 2 h or overnight (for 40 µm- and 50 µm-thick slices, respectively) at 4°C with secondary antibodies (see Table S3) in 0.01 M PBS (pH 7.4), 0.2% Triton X-100 and NDS (1%). Sections were washed in 0.01 M PBS (pH 7.4) and incubated for 20 min at room temperature with 4,6-diamidino-2-phenylindole (DAPI; 1 µg/ml) to label nuclei. Sections were washed in 0.01 M PBS (pH 7.4), then mounted on gelatine-coated slides, air dried and coverslipped with antifade mounting medium Mowiol (4-88 reagent; 475904, Calbiochem).

### Microscope acquisition and quantifications

Images of multiple immunofluorescence on tissue sections were acquired with a TCS SP5 confocal microscope (Leica Microsystems) or a Nikon microscope coupled with a computer-assisted image analysis system (Neurolucida software, MicroBrightField). Confocal *z*-stacked images used for morphometric analyses on both cellular and mitochondrial architecture were captured through the thickness of the slice (50 µm) at 0.5 µm optical steps with a 63×/1.4 NA oil immersion lens objective with an additional zoom (2×) and a resolution of 8 bit, 1024/1024 pixels and 50 Hz scan speed (1 voxel=133.6×133.6×395.2 nm; *xyz*). mitoDsRed^+^YFP^+^ newborn granule neurons residing in the upper and lower blades of the DG were used for analyses only when satisfying all the following criteria: (1) positive for doublecortin (i.e. triple DCX^+^mitoDsRed^+^YFP^+^ newborn cells); (2) bearing an apical dendrite that arborizes into the molecular cell layer; (3) showing a high and homogeneous mitoDsRed signal; and (4) including the whole dendritic morphology within the 50 µm-section thickness. Morphometric analyses of the selected DCX^+^mitoDsRed^+^YFP^+^ newborn neurons were performed by using the Simple Neurite Tracer (SNT of the Neuroanatomy toolkit) plug-in in Fiji. Perikarya size was assessed by manually drawing the largest cross-sectional area of each cell soma based on the DCX^+^YFP^+^ double staining using the polygon selection tab in Fiji. Manual counting of dendritic mitochondria number and evaluation of their length was carried out on 2D images obtained by the Max intensity projection tool applied on the optical slices that include the whole dendritic arborization. The number of dendritic mitochondria was manually analyzed by the Cell Counter and channel tool plug-ins; measurement of the dendritic mitochondrial length was carried out by drawing the length (parallel to the dendrite) of each mitochondrion using the segmented line tool in Fiji.

Analyses of the mitochondrial mass in the soma and the primary dendrite compartments were carried out as described below. First, for each cell compartment, a maximum *z*-projection of all the optical slices including the region of interest (ROI) was applied, and the ROI based on the cytoplasmic YFP^+^ signal in Fiji was drawn and measured as area in µm^2^. In particular, the soma area comprises the perikaryon area plus the cone-shaped portion of the primary dendrite directly stemming from the soma, whereas the area of the primary dendrite includes the cylindrical part of primary apical dendrite to its first ramification (see Fig. S2C). Afterwards, the channel containing the mitochondrial signal (i.e. mitoDsRed) was binarized after thresholding, and the resulting binary image was used to calculate the area covered by mitochondrial signal and expressed as a percentage of the ROI area. In addition, the MiNA toolkit developed by the Stuart laboratory (Valente et al., 2017) was used for the semi-automated analyses of the mitochondrial network features in the soma compartment.

Analysis of the mitochondrial mass in the dendritic arbor was carried out on 2D images encompassing the whole dendritic arbor: (1) we calculated the linear mitochondrial occupancy by summing the length of all the mitochondria (μm) and normalizing it over the length of the dendritic arbor (μm) and expressing it as a percentage (Fig. 3K); (2) in line with the other cellular compartments (see above), we analyzed the mitochondrial occupancy as the area covered by mitochondrial signal, but, in this case, we normalized this area (μm^2^) over the length of the dendritic arbor (μm) (Fig. S2F). The latter was used to calculate the fold change in mitochondrial occupancy in the dendritic arborization (Fig. S2G).

For the analysis of mitochondrial distribution throughout the dendritic arbor, we subdivided the dendritic arborization into segments according to the branch order and assigned a score (from 0 to 1) to each dendritic mitochondrion based on their relative position within each branch (i.e. from proximal to distal). Statistical analyses were made by considering all the branches together (Fig. 3F) or by subdividing data according to the branch orders (Fig. S2E). As *Nr2f1*-icKO neurons show decreased complexity in their dendritic arbor (absence of branch orders VI and VII that were found in Ctrl neurons), analyses were restricted from level II to level V. Moreover, the fraction of mitochondria belonging to the different branch order was calculated per animal and expressed as a percentage (Fig. 3G).

To quantify MFN2, OPA1 and OxPhos immunostaining within the soma of DCX^+^YFP^+^ neurons, confocal image *z*-stacks were captured throughout the thickness of the cell bodies of DCX^+^YFP^+^ neurons. Acquisitions were done with 0.5 µm optical step size using a 63×/1.4 NA oil immersion lens objective and by adding an additional zoom (3×) with a resolution of 8 bit, 1024/1024 pixels and 50 Hz scan speed (1 voxel=120.3×120.3×395.2 nm; *xyz*). Importantly, all the parameters (i.e. laser power, gain and offset) were kept the same among acquisitions. We developed a custom-made workflow allowing an unbiased and semi-automated quantitative analysis of the specific mitochondrial staining coverage and intensity on DCX^+^YFP^+^ neurons in Fiji. First, a maximum *z*-projection of the optical slices containing the whole soma area was applied, and the soma ROI was manually drawn based on the cytoplasmic YFP^+^ signal. The obtained ROI was applied to the channel including the naïve MFN2 signal to calculate the MFN2 staining intensity within the soma shaft compartment, expressed as the corrected total cell fluorescence (CTCF), calculated by the formula

CTCF=integrated density–(ROI×mean fluorescence of background readings). (1)

The background readings were calculated by averaging three mean fluorescence values obtained by three small ROIs (5×5 µm) without the staining (i.e. blood vessel lumen or cell nuclei close to the cell of interest). To evaluate the fractional area covered by the mitochondrial staining in the soma area, we first applied a ‘pre-processing’ step on the acquired channel including the mitochondrial signal as follows: (1) unsharp mask (radius, 2; mask, 0.4); (2) subtract background (rolling, 50); (3) enhance local contrast [i.e. Contrast Limited Adaptive Histogram Equalization (CLAHE); blocksize, 9; histogram, 256; maximum, 4; no mask; slow]; (4) median filtering (radius, 1). Afterwards, the channel containing the MFN2 staining was given a threshold (by Moments algorithm), and the binary image was cleaned outside the ROI to get the area occupied by mitochondrial staining (i.e. mitochondrial ROI) within the selected cell. Finally, we calculated the area fraction covered by mitochondrial signal on the soma area (i.e. MFN2 or OPA1 or ETC/OxPhos staining occupancy) and expressed it as a percentage. The MFN2 ROI was also applied to the naïve channel containing the mitochondrial signal to calculate the mitochondrial staining intensity, both as integrated density and as corrected total mitochondrial fluorescence (CTMF) where the ROI was the mitochondrial signal, or as the CTCF where the ROI was the soma area.

For the quantification of LAMP1 immunostaining and mitoDsRed signals within the soma of newborn YFP^+^ neurons, confocal image *z*-stacks were captured throughout the thickness of the cell bodies of YFP^+^ neurons. First, the area of the YFP^+^ signal was drawn and used to clear the signal outside of the cell per *z*-plane. Afterwards, a maximum *z*-projection of all the optical slices was applied, and total soma area was drawn based on the cytoplasmic YFP^+^ signal and measured as area in µm^2^. Then, the channel containing either LAMP1 or mitoDsRed was binarized after thresholding, and the resulting binary image was used to calculate the area covered by mitochondrial signal and expressed as a percentage of the ROI area. Finally, the area covered by both the LAMP1 signal and the mitoDsRed signal (i.e. overlapping signal) was obtained by using the ‘AND’ key on the ROI manager tool in Fiji. To further quantify the amount of LAMP1 and mitoDsRed colocalization, the Manders' coefficients was calculated by exploiting the JACoP plug-in (v2.1.4; Bolte and Cordelières, 2006) in Fiji.

To analyze morphological synaptic integration of transduced cells, confocal images of dendritic segments in the mid-third of the molecular layer from single- and double-transduced cells expressing a fluorescent marker (GFP and mitoDsRed) were obtained with a 63× glycerol objective using a Leica TCS Sp5 confocal microscope (step size, 0.4 µm; resolution, 2048×2048; 2× zoom). Length of dendrites was measured using Fiji, and number of dendritic spines was manually quantified and normalized to the dendritic length to be expressed as the number of spines/10 µm.

For all analyses, the numbers of cells, animals and mitochondria are described in the figure legends.

### Mitochondria isolation from mouse brain tissue

Mitochondria were isolated from fresh brain tissue from both WT and NR2F1 heterozygous (*Nr2f1*-HET) mice with a Mitochondria Isolation Kit for tissue (ab110168, Abcam, Cambridge, MA, USA), according to the manufacturer's instructions. Briefly, the mouse brain tissue was washed twice with the washing buffer and then suspended in mitochondria isolation buffer to be homogenized with a 2 ml Kimble dounce tissue grinder (D8938-1SET, Sigma-Aldrich) and spun at 1000 ***g*** for 10 min at 4°C; the supernatant was then transferred to a new tube and centrifuged again at 12,000 ***g*** for 15 min at 4°C. Mitochondria pellets were resuspended twice using mitochondria isolation buffer containing protease inhibitor cocktail (Halt Protease Inhibitor Single-Use Cocktail 100X; 78430, Thermo Fisher Scientific). Final mitochondria isolates were then aliquoted, stored at −80°C and subsequently used for WB analysis.

### WB analysis

Mitochondria protein concentration was determined using a Bicinchoninic Acid (BCA) Protein Assay Kit (B9643, Sigma-Aldrich, Milan, Italy). Then, 15 μg protein for each sample was loaded and separated into a 4–20% precast polyacrylamide gel (Mini-PROTEAN^®^ TGX Stain-Free; 4568096, Bio-Rad Laboratories), transferred to a supported nitrocellulose membrane (162-0093, Bio-Rad Laboratories) and blocked for 1 h at room temperature (RT) in 1× TBST (150 mM NaCl, 10 mM Tris-HCl pH 7.4, 0.1% Tween 20) containing 5% nonfat milk (1706404, Bio-Rad Laboratories). After blocking, the membrane was incubated overnight at 4°C with the primary antibodies listed in Table S4, washed and incubated with the secondary horseradish peroxidase-linked antibodies listed in Table S5. All primary antibodies were diluted in 1× TBST containing 5% bovine serum albumin (BSA) and 0.02% sodium azide, whereas all secondary antibodies were diluted in 1× TBST containing 5% nonfat milk. For protein band detection, the membrane was then incubated with a chemiluminescence substrate (Clarity Western ECL Substrate, 1705061; Clarity Max ECL Substrate, 1705062; Bio-Rad Laboratories). ECL signals were detected with a ChemiDoc™ Imaging System, and bands were quantified using Image Lab version 6.1.0 build 7 software (2020; Bio-Rad Laboratories).

### ChIP-seq

Adult neocortical tissue was acutely isolated from 2-month-old C57BL/6J mice (*n*=3 males). Animals were deeply anesthetized with an intraperitoneal injection of a mixture of tiletamine and zolazepam (40 mg/kg; Zoletil, Virbac) and xylazine (5 mg/kg; Rompun, Bayer), and the brain was removed after rapid decapitation. Neocortices from both hemispheres were microdissected and pooled together, frozen and stored at −80°C until further analysis. Around 10 mg of neural tissue was chopped with a scalpel, harvested in 5 ml of 0.01 M PBS (pH 7.4) and crosslinked by adding formaldehyde to a final concentration of 1.5%, incubated for 30 min at RT on a rotator, and quenched with 0.125 M glycine for 5 min at RT. After crosslinking, the tissue was washed twice in cold 0.01 M PBS (pH 7.4) and centrifuged at 1000 ***g*** for 5 min at 4°C. Tissue pellet was resuspended in 0.25 ml sodium dodecyl sulfate (SDS) lysis buffer (50 mM Tris-HCl pH 8.0, 1% SDS, 10 mM EDTA, anti-proteases) and incubated on a rotator for 30 min at 4°C. Afterwards, the sample was sonicated for 18 cycles on high-power setting (30 s ON, 30 s OFF) using a Bioruptor Next Gen (Diagenode) and then centrifuged at 20,000 ***g*** for 10 min at 4°C.

The isolated chromatin was diluted 10-fold with chromatin immunoprecipitation (ChIP) dilution buffer (16.7 mM Tris-HCl pH 8.0, 0.01% SDS, 1.1% Triton X-100, 1.2 mM EDTA, 167 mM NaCl) (1/10 was kept as input) and incubated with 2 µg rabbit anti-COUP-TFI/NR2F1 antibody from the Studer laboratory (Tripodi et al., 2004) overnight at 4°C on a rotator. Protein G-conjugated magnetic beads (Dynal, Thermo Fisher Scientific) were saturated with PBS/1% BSA overnight at 4°C. The next day, samples were incubated with saturated beads for 2 h at 4°C on a rotator, and subsequently washed with 1 ml cold low-salt buffer (20 mM Tris-HCl pH 8.0, 0.1% SDS, 1% Triton X-100, 2 mM EDTA, 150 mM NaCl), 1 ml cold high-salt buffer (20 mM Tris-HCl pH 8.0, 0.1% SDS, 1% Triton X-100, 2 mM EDTA, 500 mM NaCl), 1 ml cold LiCl buffer (10 mM Tris-HCl pH 8.0, 1% DOC, 250 mM LiCl, 1 mM EDTA, 1% NP-40) and twice with 1 ml cold TE buffer (10 mM Tris-HCl pH 8.0, 1 mM EDTA). The immunoprecipitated chromatin was eluted with 200 µl elution buffer (10 mM Tris-HCl pH 8.0, 1 mM EDTA, 1% SDS, 150 mM NaCl, 5 mM dithiothreitol) for 30 min at RT on a rotator, and decrosslinked at 65°C overnight. The decrosslinked DNA was purified using a QIAquick PCR Purification Kit (Qiagen) according to the manufacturer's instructions.

For genome-wide analysis of binding, sequencing libraries were constructed using an NEBNext ChIP-seq Library Prep Reagent Set for Illumina and a NextSeq 500 Illumina sequencer (New England BioLabs).

### Bioinformatic analyses

The reads from sequencing were mapped to the mouse genome (mm9 assembly) using Bowtie version 0.12.7, reporting only unique hits with up to two mismatches. The redundant reads were collapsed, and peak calling was performed using MACS version 1.4.1 (Zhang et al., 2008) with normalization for IgG ChIP at a fixed *P*-value  of  1×10^−8^. Homer version 4.11 was used to perform *de novo* motif discovery and peak annotation. A ChIP-seq peak was considered associated with a gene if it showed an overlap of −3 kb/+2 kb region around a TSS of the gene. GO enrichment analysis was performed using PANTHER Overrepresentation Test version 14 (Mi et al., 2019).

We performed a pre-rank GSEA using as ‘rank value’ the DeSeq2 statistic of gene expression in the hippocampi of adult *Nr2f1*-HET versus WT mice obtained from bulk RNA sequencing by Chen et al. (2020), and as ‘pathway’ genes bound on the promoter by NR2F1 according to the ChIP-seq. The core enrichment set includes those genes that appear in the ranked list before the point at which the running sum reaches its maximum deviation from zero (leading-edge subset) and can be interpreted as the core that accounts for the gene set's enrichment signal (Subramanian et al., 2005).

To understand how NR2F1 direct targets may work together, we used STRING to conduct a PPI analysis whereby the input gene list was (1) the complete list of nuclear genes showing NR2F1 binding on their promoters (Fig. 1) or (2) the GSEA core gene list (Fig. 4; Table S1). This analysis was done applying standard STRING defaults in the analysis (i.e. full network type; confidence level, 0.4). The resulting protein network plot was then colored with a data-driven *k*-means clustering with *k*=2 in order to visually demarcate proteins that cluster together into the mitochondrial and nuclear cell compartments for ChiP-seq data (Fig. 1) and to highlight the plethora of mitochondrial biological processes in which direct deregulated NR2F1 targets are involved (Fig. 4).

### Statistics

Data are derived from at least three different animals/group. A two-tailed unpaired Student's *t*-test was performed to compare the differences between two groups, an *F*-test of equality of variances was conducted to compare variances, and Welch's correction was applied in case of unequal variance distribution. Data distribution was tested to be normal with either the Shapiro–Wilk test (alpha, 0.05; if 3<*n*<50) or D'Agostino-Pearson (if *n*>50). When comparing two populations of data, two-tailed unpaired Student's *t*-test was used to calculate statistical significance in cases of Gaussian distribution; otherwise, the non-parametric Mann–Whitney test was used. For Sholl analysis, two-way repeated measures ANOVA with Sidak's post-hoc test was used. The confidence interval was expressed as 95% confidence. For the analysis of normalized data (i.e. fold change) a Kruskal–Wallis test followed by Dunn's post-hoc test was used. Kolmogorov–Smirnov test was carried out to compare cumulative distributions. Statistical significance was defined as follows: *P<0.05, **P<0.01 and ***P<0.001. All statistical analyses were performed using GraphPad Prism 8 software (version 8.0.2; GraphPad Software, San Diego, CA, USA). Fisher's exact test, with the Benjamini–Hochberg FDR correction for multiple testing, has been added as the default algorithm for the over-representation test for GO analyses (Mi et al., 2019).

## Supplementary Material

10.1242/dmm.049854_sup1 Supplementary information

## References

[DMM049854C1] Aimone, J. B., Li, Y., Lee, S. W., Clemenson, G. D., Deng, W. and Gage, F. H. (2014). Regulation and function of adult neurogenesis: from genes to cognition. *Physiol. Rev.* 94, 991-1026. 10.1152/physrev.00004.2014 25287858 PMC4280160

[DMM049854C2] Alfano, C., Magrinelli, E., Harb, K., Hevner, R. F. and Studer, M. (2014). Postmitotic control of sensory area specification during neocortical development. *Nat. Commun.* 5, 5632. 10.1038/ncomms6632 25476200

[DMM049854C3] Armentano, M., Filosa, A., Andolfi, G. and Studer, M. (2006). COUP-TFI is required for the formation of commissural projections in the forebrain by regulating axonal growth. *Development* 133, 4151-4162. 10.1242/dev.02600 17021036

[DMM049854C4] Armentano, M., Chou, S. J., Srubek Tomassy, G., Leingärtner, A., O'Leary, D. D. M. and Studer, M. (2007). COUP-TFI regulates the balance of cortical patterning between frontal/motor and sensory areas. *Nat. Neurosci.* 10, 1277-1286. 10.1038/nn1958 17828260

[DMM049854C5] Artegiani, B., Lyubimova, A., Muraro, M., van Es, J. H., van Oudenaarden, A. and Clevers, H. (2017). A single-cell RNA sequencing study reveals cellular and molecular dynamics of the hippocampal neurogenic niche. *Cell Rep* 21, 3271-3284. 10.1016/j.celrep.2017.11.050 29241552

[DMM049854C6] Bapat, O., Purimetla, T., Kruessel, S., Thum, C., Rupprecht, F., Shah, M., Langer, J. D. and Rangaraju, V. (2023). VAP spatially stabilizes dendritic mitochondria to locally fuel synaptic plasticity*. bioRxiv*. 10.1101/2023.01.16.524245 PMC10766606 38177103

[DMM049854C7] Beckervordersandforth, R. (2017). Mitochondrial metabolism-mediated regulation of adult neurogenesis. *Brain Plast* 3, 73-87. 10.3233/BPL-170044 29765861 PMC5928529

[DMM049854C8] Beckervordersandforth, R., Ebert, B., Schäffner, I., Moss, J., Fiebig, C., Shin, J., Moore, D. L., Ghosh, L., Trinchero, M. F., Stockburger, C. et al. (2017). Role of mitochondrial metabolism in the control of early lineage progression and aging phenotypes in adult hippocampal neurogenesis. *Neuron* 93, 560-573.e6. 10.1016/j.neuron.2016.12.017 28111078 PMC5300896

[DMM049854C9] Bertacchi, M., Gruart, A., Kaimakis, P., Allet, C., Serra, L., Giacobini, P., Delgado-García, J. M., Bovolenta, P. and Studer, M. (2019a). Mouse Nr2f1 haploinsufficiency unveils new pathological mechanisms of a human optic atrophy syndrome. *EMBO Mol Med* 11, 1-18. 10.15252/emmm.201910291 PMC6685104 31318166

[DMM049854C10] Bertacchi, M., Parisot, J. and Studer, M. (2019b). The pleiotropic transcriptional regulator COUP-TFI plays multiple roles in neural development and disease. *Brain Res.* 1705, 75-94. 10.1016/j.brainres.2018.04.024 29709504

[DMM049854C11] Bertacchi, M., Romano, A. L., Loubat, A., Tran Mau-Them, F., Willems, M., Faivre, L., van Kien, K., Perrin, P., Devillard, L., Sorlin, F. (2020). NR2F1 regulates regional progenitor dynamics in the mouse neocortex and cortical gyrification in BBSOAS patients. *EMBO J.* 39, e104163. 10.15252/embj.2019104163 32484994 PMC7327499

[DMM049854C12] Bhattacharyya, A., McMillan, E., Chen, S. I., Wallace, K. and Svendsen, C. N. (2009). A critical period in cortical interneuron neurogenesis in Down syndrome revealed by human neural progenitor cells. *Dev. Neurosci.* 31, 497-510. 10.1159/000236899 19738365 PMC2818457

[DMM049854C13] Billiet, B., Amati-Bonneau, P., Desquiret-Dumas, V., Guehlouz, K., Milea, D., Gohier, P., Lenaers, G., Mirebeau-Prunier, D., den Dunnen, J. T., Reynier, P. et al. (2021). NR2F1 database: 112 variants and 84 patients support refining the clinical synopsis of Bosch–Boonstra–Schaaf optic atrophy syndrome. *Hum. Mutat.* 43, 128-142. 10.1002/humu.24305 34837429

[DMM049854C14] Bolte, S. and Cordelières, F. P. (2006). A guided tour into subcellular colocalization analysis in light microscopy. *J. Microsc.* 224, 213-232. 10.1111/j.1365-2818.2006.01706.x 17210054

[DMM049854C15] Bond, A. M., Ming, G. L. and Song, H. (2015). Adult mammalian neural stem cells and neurogenesis: five decades later. *Cell Stem Cell* 17, 385-395. 10.1016/j.stem.2015.09.003 26431181 PMC4683085

[DMM049854C16] Bonzano, S., Crisci, I., Podlesny-Drabiniok, A., Rolando, C., Krezel, W., Studer, M. and De Marchis, S. (2018). Neuron-astroglia cell fate decision in the adult mouse hippocampal neurogenic niche is cell-intrinsically controlled by COUP-TFI In Vivo. *Cell Rep* 24, 329-341. 10.1016/j.celrep.2018.06.044 29996095

[DMM049854C17] Bosch, D. G. M., Boonstra, F. N., Gonzaga-Jauregui, C., Xu, M., De Ligt, J., Jhangiani, S., Wiszniewski, W., Muzny, D. M., Yntema, H. G., Pfundt, R. et al. (2014). NR2F1 mutations cause optic atrophy with intellectual disability. *Am. J. Hum. Genet.* 94, 303-309. 10.1016/j.ajhg.2014.01.002 24462372 PMC3928641

[DMM049854C18] Bosch, D. G. M., Boonstra, F. N., De Leeuw, N., Pfundt, R., Nillesen, W. M., De Ligt, J., Gilissen, C., Jhangiani, S., Lupski, J. R., Cremers, F. P. M. et al. (2016). Novel genetic causes for cerebral visual impairment. *Eur. J. Hum. Genet.* 24, 660-665. 10.1038/ejhg.2015.186 26350515 PMC4930090

[DMM049854C19] Bovetti, S., Bonzano, S., Garzotto, D., Giannelli, S. G., Iannielli, A., Armentano, M., Studer, M. and De Marchis, S. (2013). COUP-TFI controls activity-dependent tyrosine hydroxylase expression in adult dopaminergic olfactory bulb interneurons. *Development* 140, 4850-4859. 10.1242/dev.089961 24227652

[DMM049854C77] Calvo, S. E., Clauser, K. R. and Mootha, V. K. (2016). MitoCarta2.0: an updated inventory of mammalian mitochondrial proteins. *Nucleic Acids Res.* 44, D1251-D1257. 10.1093/nar/gkv1003 26450961 PMC4702768

[DMM049854C20] Chan, D. C. (2020). Mitochondrial dynamics and its involvement in disease. *Annu. Rev. Pathol. Mech. Dis.* 15, 235-259. 10.1146/annurev-pathmechdis-012419-032711 31585519

[DMM049854C21] Chen, C. A., Wang, W., Pedersen, S. E., Raman, A., Seymour, M. L., Ruiz, F. R., Xia, A., Van Der Heijden, M. E., Wang, L., Yin, J. et al. (2020). Nr2f1 heterozygous knockout mice recapitulate neurological phenotypes of Bosch-Boonstra-Schaaf optic atrophy syndrome and show impaired hippocampal synaptic plasticity. *Hum. Mol. Genet.* 29, 705-715. 10.1093/hmg/ddz233 31600777 PMC7104670

[DMM049854C22] Chen, C. A., Bosch, D. G. M., Cho, M. T., Rosenfeld, J. A., Shinawi, M., Lewis, R. A., Mann, J., Jayakar, P., Payne, K., Walsh, L. et al. (2016). The expanding clinical phenotype of Bosch-Boonstra-Schaaf optic atrophy syndrome: 20 new cases and possible genotype-phenotype correlations. *Genet. Med.* 18, 1143-1150. 10.1038/gim.2016.18 26986877

[DMM049854C23] Course, M. M. and Wang, X. (2016). Transporting mitochondria in neurons. *F1000Research* 5, 1735. 10.12688/f1000research.7864.1 PMC4955021 27508065

[DMM049854C78] Dickey, A. S. and Strack, S. (2011). PKA/AKAP1 and PP2A/Bβ2 regulate neuronal morphogenesis via Drp1 phosphorylation and mitochondrial bioenergetics. *J. Neurosci.* 31, 15716-15726. 10.1523/JNEUROSCI.3159-11.2011 22049414 PMC3328351

[DMM049854C25] Divakaruni, S. S., Van Dyke, A. M., Chandra, R., LeGates, T. A., Contreras, M., Dharmasri, P. A., Higgs, H. N., Lobo, M. K., Thompson, S. M. and Blanpied, T. A. (2018). Long-term potentiation requires a rapid burst of dendritic mitochondrial fission during induction. *Neuron* 100, 860-875.e7. 10.1016/j.neuron.2018.09.025 30318410 PMC6483400

[DMM049854C26] Faits, M. C., Zhang, C., Soto, F. and Kerschensteiner, D. (2016). Dendritic mitochondria reach stable positions during circuit development. *Elife* 5, 1-17. 10.7554/eLife.11583 PMC4749546 26742087

[DMM049854C27] Fang, D., Yan, S., Yu, Q., Chen, D. and Yan, S. S. (2016). Mfn2 is required for mitochondrial development and synapse formation in human induced pluripotent stem cells/hiPSC derived cortical neurons. *Sci. Rep.* 6, 1-13. 10.1038/s41598-016-0001-8 27535796 PMC4989148

[DMM049854C28] Frye, R. E. (2020). Mitochondrial dysfunction in autism spectrum disorder: unique abnormalities and targeted treatments. *Semin. Pediatr. Neurol.* 35, 100829. 10.1016/j.spen.2020.100829 32892956

[DMM049854C29] Giacomello, M., Pyakurel, A., Glytsou, C. and Scorrano, L. (2020). The cell biology of mitochondrial membrane dynamics. *Nat. Rev. Mol. Cell Biol.* 21, 204-224. 10.1038/s41580-020-0210-7 32071438

[DMM049854C30] Gonçalves, J. T., Schafer, S. T. and Gage, F. H. (2016). Adult neurogenesis in the hippocampus: from stem cells to behavior. *Cell* 167, 897-914. 10.1016/j.cell.2016.10.021 27814520

[DMM049854C31] Halevy, T., Biancotti, J. C., Yanuka, O., Golan-Lev, T. and Benvenisty, N. (2016). Molecular characterization of Down syndrome embryonic stem cells reveals a role for RUNX1 in neural differentiation. *Stem Cell Reports* 7, 777-786. 10.1016/j.stemcr.2016.08.003 27618722 PMC5063584

[DMM049854C32] Han, S., Nandy, P., Austria, Q., Siedlak, S. L., Torres, S., Fujioka, H., Wang, W. and Zhu, X. (2020). Mfn2 ablation in the adult mouse hippocampus and cortex causes neuronal death. *Cells* 9, 116. 10.3390/cells9010116 31947766 PMC7017224

[DMM049854C33] Hobbs, M. M., Wolters, W. C., Rayapati, A. O. and Kahn, D. A. (2020). Bosch-Boonstra-Schaaf optic atrophy syndrome presenting as new-onset psychosis in a 32-year-old man: a case report and literature review. *J. Psychiatr. Pract.* 26, 58-62. 10.1097/PRA.0000000000000440 31913971

[DMM049854C76] Hock, M. B. and Kralli, A. (2009). Transcriptional control of mitochondrial biogenesis and function. *Annu. Rev. Physiol.* 71, 177-203. 10.1146/annurev.physiol.010908.163119 19575678

[DMM049854C34] Iwata, R., Casimir, P., Erkol, E., Boubakar, L., Planque, M., Gallego López, I. M., Ditkowska, M., Gaspariunaite, V., Beckers, S., Remans, D. et al. (2023). Mitochondria metabolism sets the species-specific tempo of neuronal development. *Science* 379, eabn4705. 10.1126/science.abn4705 36705539

[DMM049854C35] Jagasia, R., Steib, K., Englberger, E., Herold, S., Faus-Kessler, T., Saxe, M., Gage, F. H., Song, H. and Lie, D. C. (2009). GABA-cAMP response element-binding protein signaling regulates maturation and survival of newly generated neurons in the adult hippocampus. *J. Neurosci.* 29, 7966-7977. 10.1523/JNEUROSCI.1054-09.2009 19553437 PMC2776747

[DMM049854C36] Jurcau, A. (2021). Insights into the pathogenesis of neurodegenerative diseases: Focus on mitochondrial dysfunction and oxidative stress. *Int. J. Mol. Sci.* 22, 11847. 10.3390/ijms222111847 34769277 PMC8584731

[DMM049854C37] Jurkute, N., Bertacchi, M., Arno, G., Tocco, C., Kim, U. S., Kruszewski, A. M., Avery, R. A., Bedoukian, E. C., Han, J., Ahn, S. J., et al. (2021). Pathogenic NR2F1 variants cause a developmental ocular phenotype recapitulated in a mutant mouse model. *Brain Commun* 3, 1-21. 10.1093/braincomms/fcab162 PMC8397830 34466801

[DMM049854C38] Kaiwar, C., Zimmermann, M. T., Ferber, M. J., Niu, Z., Urrutia, R. A., Klee, E. W. and Babovic-Vuksanovic, D. (2017). Novel NR2F1 variants likely disrupt DNA binding: molecular modeling in two cases, review of published cases, genotype-phenotype correlation, and phenotypic expansion of the Bosch-Boonstra-Schaaf optic atrophy syndrome. *Cold Spring Harb Mol case Stud* 3, a002162. 10.1101/mcs.a002162 28963436 PMC5701304

[DMM049854C39] Khacho, M., Clark, A., Svoboda, D. S., Azzi, J., MacLaurin, J. G., Meghaizel, C., Sesaki, H., Lagace, D. C., Germain, M., Harper, M. E. et al. (2016). Mitochondrial dynamics impacts stem cell identity and fate decisions by regulating a nuclear transcriptional program. *Cell Stem Cell* 19, 232-247. 10.1016/j.stem.2016.04.015 27237737

[DMM049854C40] Khacho, M., Harris, R. and Slack, R. S. (2019). Mitochondria as central regulators of neural stem cell fate and cognitive function. *Nat. Rev. Neurosci.* 20, 34-48. 10.1038/s41583-018-0091-3 30464208

[DMM049854C41] Kimura, T. and Murakami, F. (2014). Evidence that dendritic mitochondria negatively regulate dendritic branching in pyramidal neurons in the neocortex. *J. Neurosci.* 34, 6938-6951. 10.1523/JNEUROSCI.5095-13.2014 24828647 PMC6608104

[DMM049854C42] Lenaers, G., Neutzner, A., Le Dantec, Y., Jüschke, C., Xiao, T., Decembrini, S., Swirski, S., Kieninger, S., Agca, C., Kim, U. S. et al. (2021). Dominant optic atrophy: Culprit mitochondria in the optic nerve. *Prog. Retin. Eye Res.* 83, 100935. 10.1016/j.preteyeres.2020.100935 33340656

[DMM049854C43] Li, Z., Okamoto, K., Hayashi, Y. and Sheng, M. (2004). The importance of dendritic mitochondria in the morphogenesis and plasticity of spines and synapses. *Cell* 119, 873-887. 10.1016/j.cell.2004.11.003 15607982

[DMM049854C44] Liang, G., Lin, J. C. Y., Wei, V., Yoo, C., Cheng, J. C., Nguyen, C. T., Weisenberger, D. J., Egger, G., Takai, D., Gonzales, F. A. et al. (2004). Distinct localization of histone H3 acetylation and H3-K4 methylation to the transcription start sites in the human genome. *Proc Natl Acad Sci* 101, 7357-7362. 10.1073/pnas.0401866101 15123803 PMC409923

[DMM049854C45] López-Doménech, G., Higgs, N. F., Vaccaro, V., Ros, H., Arancibia-Càrcamo, I. L., MacAskill, A. F. and Kittler, J. T. (2016). Loss of dendritic complexity precedes neurodegeneration in a mouse model with disrupted mitochondrial distribution in mature dendrites. *Cell Rep* 17, 317-327. 10.1016/j.celrep.2016.09.004 27705781 PMC5067282

[DMM049854C46] Martín-Hernández, E., Rodríguez-García, M. E., Chen, C. A., Cotrina-Vinagre, F. J., Carnicero-Rodríguez, P., Bellusci, M., Schaaf, C. P. and Martínez-Azorín, F. (2018). Mitochondrial involvement in a Bosch-Boonstra-Schaaf optic atrophy syndrome patient with a novel de novo NR2F1 gene mutation. *J. Hum. Genet.* 63, 525-528. 10.1038/s10038-017-0398-3 29410510

[DMM049854C47] Mi, H., Muruganujan, A., Huang, X., Ebert, D., Mills, C., Guo, X. and Thomas, P. D. (2019). Protocol Update for large-scale genome and gene function analysis with the PANTHER classification system (v.14.0). *Nat. Protoc.* 14, 703-721. 10.1038/s41596-019-0128-8 30804569 PMC6519457

[DMM049854C48] Misgeld, T. and Schwarz, T. L. (2017). Mitostasis in neurons: maintaining mitochondria in an extended cellular architecture. *Neuron* 96, 651-666. 10.1016/j.neuron.2017.09.055 29096078 PMC5687842

[DMM049854C49] Montemayor, C., Montemayor, O. A., Ridgeway, A., Lin, F., Wheeler, D. A., Pletcher, S. D. and Pereira, F. A. (2010). Genome-wide analysis of binding sites and direct target genes of the orphan nuclear receptor NR2F1/COUP-TFI. *PLoS One* 5, e8910. 10.1371/journal.pone.0008910 20111703 PMC2811727

[DMM049854C50] Monzio Compagnoni, G., Di Fonzo, A., Corti, S., Comi, G. P., Bresolin, N. and Masliah, E. (2020). The role of mitochondria in neurodegenerative diseases: the lesson from Alzheimer's disease and Parkinson's disease. *Mol. Neurobiol.* 57, 2959-2980. 10.1007/s12035-020-01926-1 32445085 PMC9047992

[DMM049854C51] Naka, H., Nakamura, S., Shimazaki, T. and Okano, H. (2008). Requirement for COUP-TFI and II in the temporal specification of neural stem cells in CNS development. *Nat. Neurosci.* 11, 1014-1023. 10.1038/nn.2168 19160499

[DMM049854C52] Pfanner, N., Warscheid, B. and Wiedemann, N. (2019). Mitochondrial proteins: from biogenesis to functional networks. *Nat. Rev. Mol. Cell Biol* 20, 267-284. 10.1038/s41580-018-0092-0 30626975 PMC6684368

[DMM049854C53] Qiu, Y., Krishnan, V., Zeng, Z., Gilbert, D. J., Copeland, N. G., Gibson, L., Yang-Feng, T., Jenkins, N. A., Tsai, M. J. and Tsai, S. Y. (1995). Isolation, characterization, and chromosomal localization of mouse and human COUP-TF I and II Genes. *Genomics* 29, 240-246. 10.1006/geno.1995.1237 8530078

[DMM049854C54] Quintana-Cabrera, R. and Scorrano, L. (2023). Determinants and outcomes of mitochondrial dynamics. *Mol. Cell* 83, 857-876. 10.1016/j.molcel.2023.02.012 36889315

[DMM049854C55] Rada-Iglesias, A., Bajpai, R., Prescott, S., Brugmann, S. A., Swigut, T. and Wysocka, J. (2012). Epigenomic annotation of enhancers predicts transcriptional regulators of human neural crest. *Cell Stem Cell* 11, 633-648. 10.1016/j.stem.2012.07.006 22981823 PMC3751405

[DMM049854C56] Rangaraju, V., Lauterbach, M. and Schuman, E. M. (2019a). Spatially stable mitochondrial compartments fuel local translation during plasticity. *Cell* 176, 73-84.e15. 10.1016/j.cell.2018.12.013 30612742

[DMM049854C57] Rangaraju, V., Lewis, T. L., Hirabayashi, Y., Bergami, M., Motori, E., Cartoni, R., Kwon, S. K. and Courchet, J. (2019b). Pleiotropic mitochondria: the influence of mitochondria on neuronal development and disease. *J. Neurosci.* 39, 8200-8208. 10.1523/JNEUROSCI.1157-19.2019 31619488 PMC6794931

[DMM049854C58] Rath, S., Sharma, R., Gupta, R., Ast, T., Chan, C., Durham, T. J., Goodman, R. P., Grabarek, Z., Haas, M. E., Hung, W. H. W. et al. (2021). MitoCarta3.0: an updated mitochondrial proteome now with sub-organelle localization and pathway annotations. *Nucleic Acids Res. J* 49, D1541-D1547. 10.1093/nar/gkaa1011 PMC7778944 33174596

[DMM049854C59] Rech, M. E., McCarthy, J. M., Chen, C. A., Edmond, J. C., Shah, V. S., Bosch, D. G. M., Berry, G. T., Williams, L., Madan-Khetarpal, S., Niyazov, D. et al. (2020). Phenotypic expansion of Bosch–Boonstra–Schaaf optic atrophy syndrome and further evidence for genotype–phenotype correlations. *Am J Med Genet Part A* 182, 1426-1437. 10.1002/ajmg.a.61580 32275123

[DMM049854C60] Rojas-Charry, L., Nardi, L., Methner, A. and Schmeisser, M. J. (2021). Abnormalities of synaptic mitochondria in autism spectrum disorder and related neurodevelopmental disorders. *J. Mol. Med.* 99, 161-178. 10.1007/s00109-020-02018-2 33340060 PMC7819932

[DMM049854C61] Steib, K., Schäffner, I., Jagasia, R., Ebert, B. and Lie, D. C. (2014). Mitochondria modify exercise-induced development of stem cell-derived neurons in the adult brain. *J. Neurosci.* 34, 6624-6633. 10.1523/JNEUROSCI.4972-13.2014 24806687 PMC6608139

[DMM049854C62] Subramanian, A., Tamayo, P., Mootha, V. K., Mukherjee, S., Ebert, B. L., Gillette, M. A., Paulovich, A., Pomeroy, S. L., Golub, T. R., Lander, E. S. et al. (2005). Gene set enrichment analysis: a knowledge-based approach for interpreting genome-wide expression profiles. *Proc Natl Acad Sci* 102, 15545-15550. 10.1073/pnas.0506580102 16199517 PMC1239896

[DMM049854C63] Szklarczyk, D., Gable, A. L., Nastou, K. C., Lyon, D., Kirsch, R., Pyysalo, S., Doncheva, N. T., Legeay, M., Fang, T., Bork, P. et al. (2021). The STRING database in 2021: customizable protein-protein networks, and functional characterization of user-uploaded gene/measurement sets. *Nucleic Acids Res.* 49, D605-D612. 10.1093/nar/gkaa1074 33237311 PMC7779004

[DMM049854C64] Tashiro, A., Makino, H. and Gage, F. H. (2007). Experience-specific functional modification of the dentate gyrus through adult neurogenesis: a critical period during an immature stage. *J. Neurosci.* 27, 3252-3259. 10.1523/JNEUROSCI.4941-06.2007 17376985 PMC6672473

[DMM049854C65] Tocco, C., Bertacchi, M. and Studer, M. (2021). Structural and functional aspects of the neurodevelopmental Gene NR2F1: from animal models to human pathology. *Front Mol Neurosci* 14, 767965. 10.3389/fnmol.2021.767965 34975398 PMC8715095

[DMM049854C66] Tripodi, M., Filosa, A., Armentano, M. and Studer, M. (2004). The COUP-TF nuclear receptors regulate cell migration in the mammalian basal forebrain. *Development* 131, 6119-6129. 10.1242/dev.01530 15548577

[DMM049854C67] Valente, A. J., Maddalena, L. A., Robb, E. L., Moradi, F. and Stuart, J. A. (2017). A simple ImageJ macro tool for analyzing mitochondrial network morphology in mammalian cell culture. *Acta Histochem.* 119, 315-326. 10.1016/j.acthis.2017.03.001 28314612

[DMM049854C68] Valenti, D., de Bari, L., De Filippis, B., Henrion-Caude, A. and Vacca, R. A. (2014). Mitochondrial dysfunction as a central actor in intellectual disability-related diseases: an overview of Down syndrome, autism, Fragile X and Rett syndrome. *Neurosci. Biobehav. Rev.* 46, 202-217. 10.1016/j.neubiorev.2014.01.012 24548784

[DMM049854C69] Veyrac, A., Gros, A., Bruel-Jungerman, E., Rochefort, C., Kleine Borgmann, F. B., Jessberger, S. and Laroche, S. (2013). *Zif268*/*egr1* gene controls the selection, maturation and functional integration of adult hippocampal newborn neurons by learning. *Proc Natl Acad Sci* 110, 7062-7067. 10.1073/pnas.1220558110 23569253 PMC3637756

[DMM049854C70] Walter, J., Bolognin, S., Poovathingal, S. K., Magni, S., Gérard, D., Antony, P. M. A., Nickels, S. L., Salamanca, L., Berger, E., Smits, L. M. et al. (2021). The Parkinson's-disease-associated mutation LRRK2-G2019S alters dopaminergic differentiation dynamics via NR2F1. *Cell Rep* 37, 109864. 10.1016/j.celrep.2021.109864 34686322

[DMM049854C71] Wu, S. P., Lee, D. K., DeMayo, F. J., Tsai, S. Y. and Tsai, M. J. (2010). Generation of ES cells for conditional expression of nuclear receptors and coregulators in vivo. *Mol. Endocrinol.* 24, 1297-1304. 10.1210/me.2010-0068 20382891 PMC2875802

[DMM049854C72] Zhang, Y., Liu, T., Meyer, C. A., Eeckhoute, J., Johnson, D. S., Bernstein, B. E., Nussbaum, C., Myers, R. M., Brown, M., Li, W. et al. (2008). Model-based analysis of ChIP-Seq (MACS). *Genome Biol.* 9, R137. 10.1186/gb-2008-9-9-r137 18798982 PMC2592715

[DMM049854C73] Zhao, C., Teng, E. M., Summers, R. G., Ming, G. L. and Gage, F. H. (2006). Distinct morphological stages of dentate granule neuron maturation in the adult mouse hippocampus. *J. Neurosci.* 26, 3-11. 10.1523/JNEUROSCI.3648-05.2006 16399667 PMC6674324

[DMM049854C74] Zheng, J., Li, H. L., Tian, N., Liu, F., Wang, L., Yin, Y., Yue, L., Ma, L., Wan, Y. and Wang, J. Z. (2020). Interneuron accumulation of phosphorylated tau impairs adult hippocampal neurogenesis by suppressing GABAergic transmission. *Cell Stem Cell* 26, 331-345.e6. 10.1016/j.stem.2019.12.015 31978364

[DMM049854C75] Zsurka, G. and Kunz, W. S. (2015). Mitochondrial dysfunction and seizures: the neuronal energy crisis. *Lancet Neurol.* 14, 956-966. 10.1016/S1474-4422(15)00148-9 26293567

