# Supplementary material for: NR2F1 shapes mitochondria in the mouse brain, providing new insights into Bosch-Boonstra-Schaaf optic atrophy syndrome
Source: Dis Model Mech. 2023 Jun 26;16(6):dmm049854. doi: 10.1242/dmm.049854 (PMC10309583; doi:10.1242/dmm.049854)
Supplement: Supplementary information [file dmm-16-049854-s1.pdf]

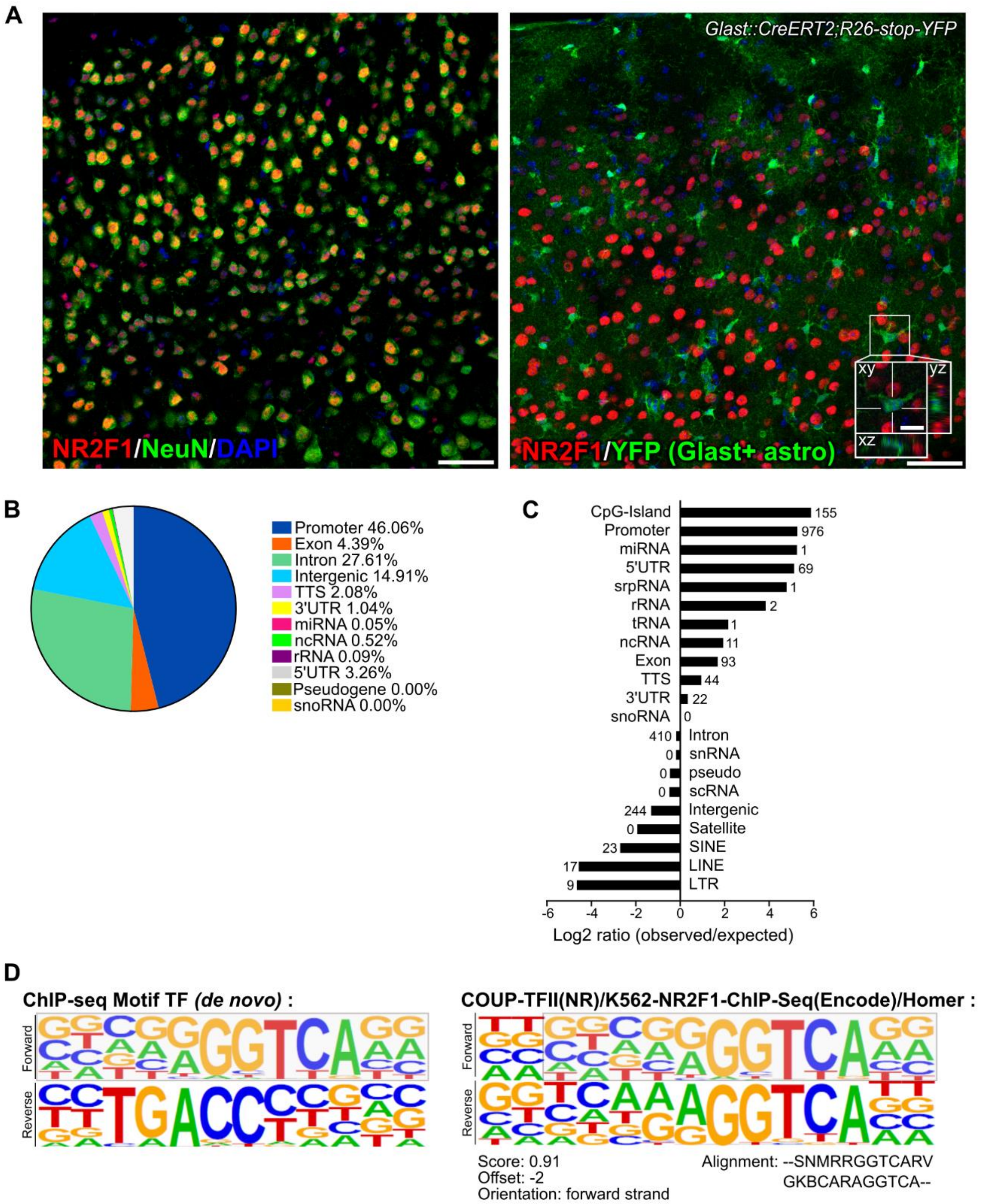

Fig. S1. Related to Figure 1. A. Representative confocal images showing NR2F1 immunofluorescence in the neuronal population within the adult mouse neocortex. The vast majority of NR2F1+ nuclei belongs to the neuronal population, as revealed by the high colocalization of NR2F1 immunofluorescence with the neuronal marker NeuN (left) and no co-expression within the recombined astrocytic population labelled by YFP in *Glast::CreERT2;Rosa26-lox-stop-lox-YFP* mice following a chase of two weeks after tamoxifen administration (right). **B**. Pie chart illustrating the relative distribution (expressed

Disease Models & Mechanisms • Supplementary information

as percentage) of the 2119 NR2F1-bound sequences. **C.** Graph reporting the logarithmic ratio between the observed genomic NR2F1-bound peaks versus the expected ones. Numbers in each line represent the quantity of annotated peaks/annotation category. **D.** Raw data showing the *de novo* motif revealed by ChIP-seq for NR2F1 in the adult brain and one of the best matches with known ChIP-seq database. Scale bars: a,b, 50 µm (low magnification); b, 10 µm (higher magnification with resliced).

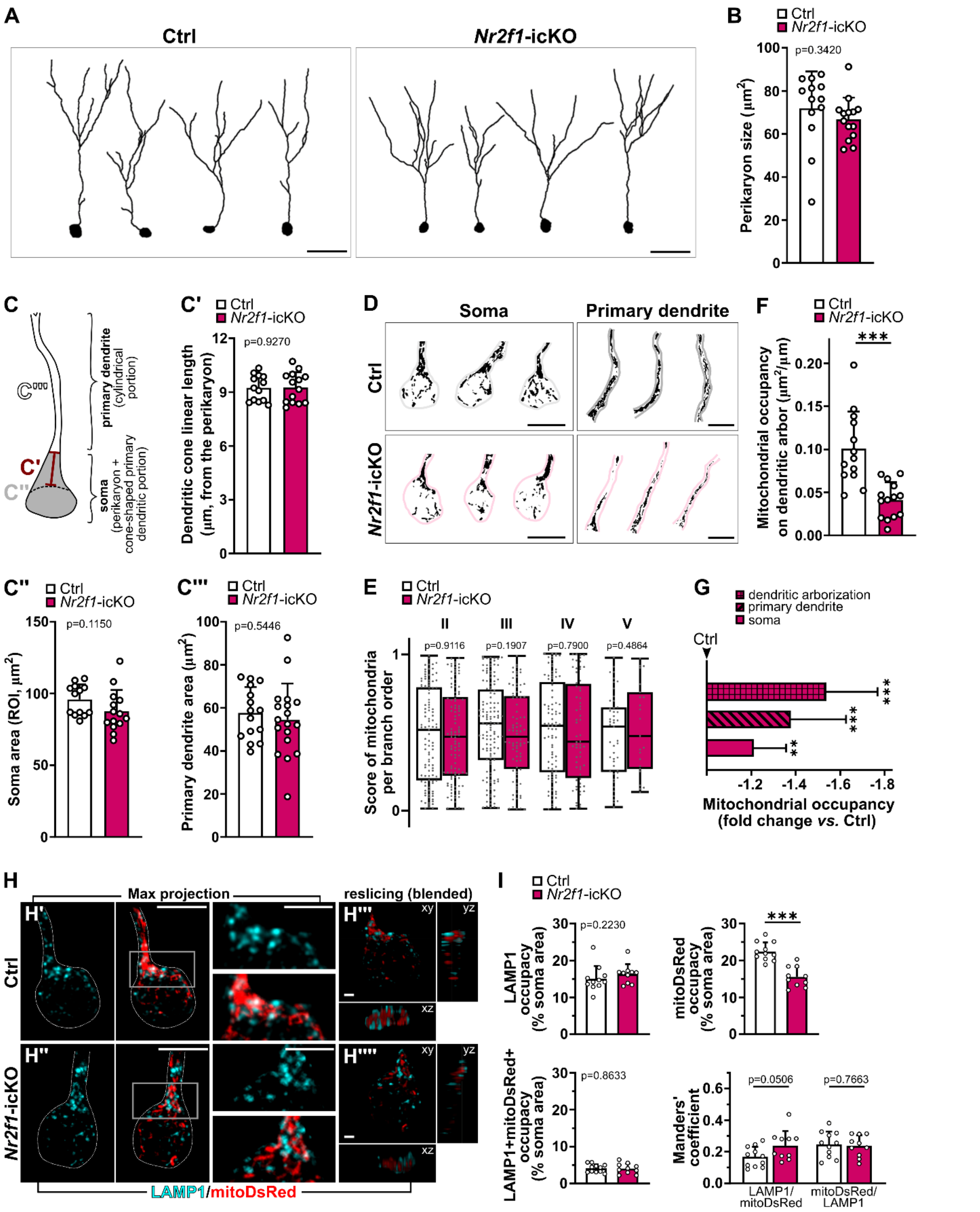

Fig. S2. Related to Figure 2 and 3. A. Representative reconstruction of triple labeled mitoDsRed+DCX+YFP+ DG newborn neurons at 17dpi and used for the morphological analyses. **B.** Histogram reporting no difference in the perikarya of control vs. NR2F1-deficient mitoDsRed+DCX+YFP+ newborn neurons. Unpaired Student's t-test. n=13 and n=14 cells out of 3 animals per genotypes and at least 3 cells/animals were used for each analysis. **C.** Drawing showing

Disease Models & Mechanisms • Supplementary information

the ROIs used for the quantifications of mitochondrial occupancy and quantified in C', C'' and C'''. **C'-C'''.** Quantification of the morphometric parameters used to calculate the mitochondrial occupancy. Student's unpaired t test, exact p-values in the graphs. n=13 Ctrl and n=14 *Nr2f1*-icKO cells out of 3 animals per genotype and at least 4 cells/animal. Data are shown as mean±SD. Statistics are included in the graphs. **D.** Representative binarized images showing the mitoDsRed+ mitochondria (black) within the somata (left) and primary dendrites (right) in control and NR2F1-deficient mitoDsRed+DCX+YFP+ newborn neurons. **F.** Graph reporting the mitochondrial occupancy expressed as the ratio of the area occupied by mitochondria in the dendritic arborization (expressed in um^2) over the length of the dendritic arborization (expressed in um). Unpaired Student's t test, p=0.0003. **E.** Quantification of the distribution of dendritic mitochondria throughout the different branch orders (from II to V); n= 379 mitochondria in Ctrl neurons; n=308 mitochondria in *Nr2f1*-icKO neurons. Mann Whitney p-values are included in the graphs. Each dot represents a mitochondrion. **G.** Histogram showing the reduction (expressed as fold change *versus* Ctrl) in the mitochondrial mass within the different subcellular compartments in NR2F1-depleted mitoDdRed+DCX+YFP+ cells compared to the one measured in the compartments of the control cells. Statistics (asterisks) were obtained by Mann Whitney test of fold change values obtained from *Nr2f1*-icKO cells and normalized to control values (*Nr2f1*-icKO *vs.* Ctrl): soma, p=0.0047; primary dendrite, p=0.0002; dendritic arborization, p<0.0001. **H.** Representative confocal images of segmented mitoDsRed+ adult-born neurons immunolabeled for the lysosome membrane protein LAMP1 (cyan) in the DG of Ctrl (top) and *Nr2f1*-icKO (bottom) mice (experimental paradigm in Figure 2A'). Max z-projection images are shown in H' and H'' and magnified to better appreciate mitoDsRed and LAMP1 overlapping stainings as well as the area covered by each marker. Resliced images in H'''and H'''' clearly show high juxtaposition of the two stainings into the z axis, but no obvious evidence of mitophagic events (i.e., mitoDsRed+ signal engulfment by LAMP1+ vesicles) in those neurons of both genotypes. **I.** Graph reporting the lysosomal LAMP1+ and the mitochondrial mitoDsRed+ occupancy expressed as the percentage of the soma area covered by each signal separately (top left and top right respectively) or by double LAMP1+mitoDsRed+ signal (bottom left). Double LAMP1+mitoDsRed+ signal was also expressed as the Manders' coefficient (bottom right) Mann Whitney p-values are included in the graphs of the % of the soma area; unpaired Student's t-test is included in the graph of the Manders' coefficient. For the analysis in I, n=11 (Ctrl) and 10 (*Nr2f1*-icKO) out of 3 animals per genotypes and at least 3 cells/animals were used. Scale bars: A, 30 µm; D, 10 µm; H',H'', 10 µm (low magnification), 5 µm (high magnification); H''',H'''', 1 µm. *p<0.05, **p<0.01, ***p<0.001.

Disease Models & Mechanisms • Supplementary information

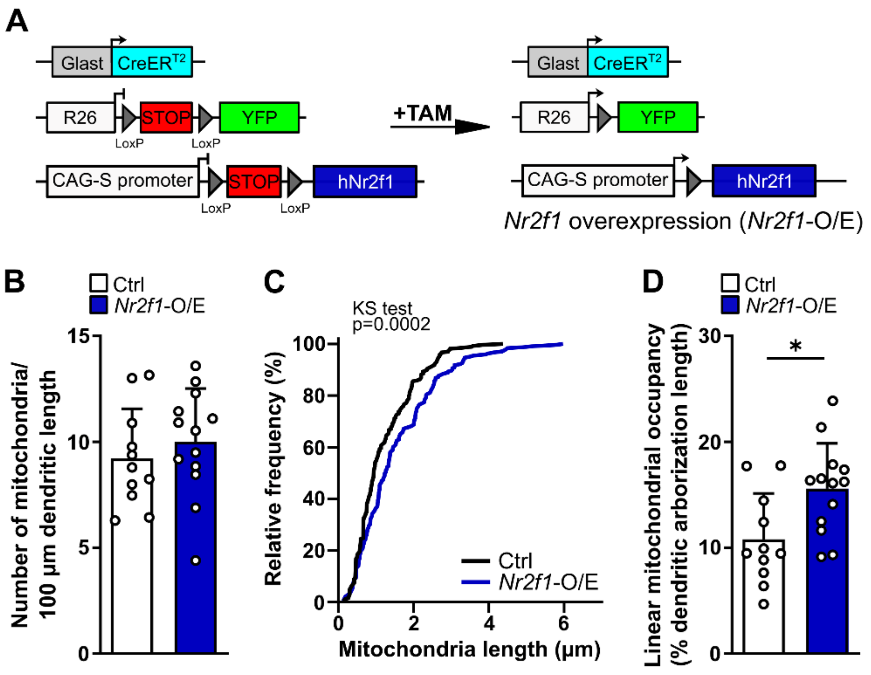

Fig. S3. A. Overview of the Cre-mediated gene rearrangements of Rosa26 loci and the transgene *CAG-loxP-stop-LoxP-hNR2F1* to induce *Nr2f1* overexpression (O/E) in the Glast lineage. Experimental design is the same used in Figure 2A'. **B.** Histogram showing the linear density of dendritic mitochondria obtained by normalizing the total number of dendritic mitochondria to the total dendritic arborization length in both Ctrl and *Nr2f1*-O/E newborn neurons. Unpaired Student's t test, p=0.4434. **C.** Cumulative frequency distribution for the length of dendritic mitochondria in control vs. *Nr2f1* overexpressing mitoDsRed+DCX+YFP+ adult-born neurons. Kolmogoror-Smirnov test, p<0.0002. **D.** Graph reporting the mitochondrial occupancy expressed as the percentage of dendritic length covered by the mitoDsRed signal. Mann Whitney test, p=0.0301. Data are shown as mean±SD. Each dot represents a cell. *p<0.05.

Disease Models & Mechanisms • Supplementary information

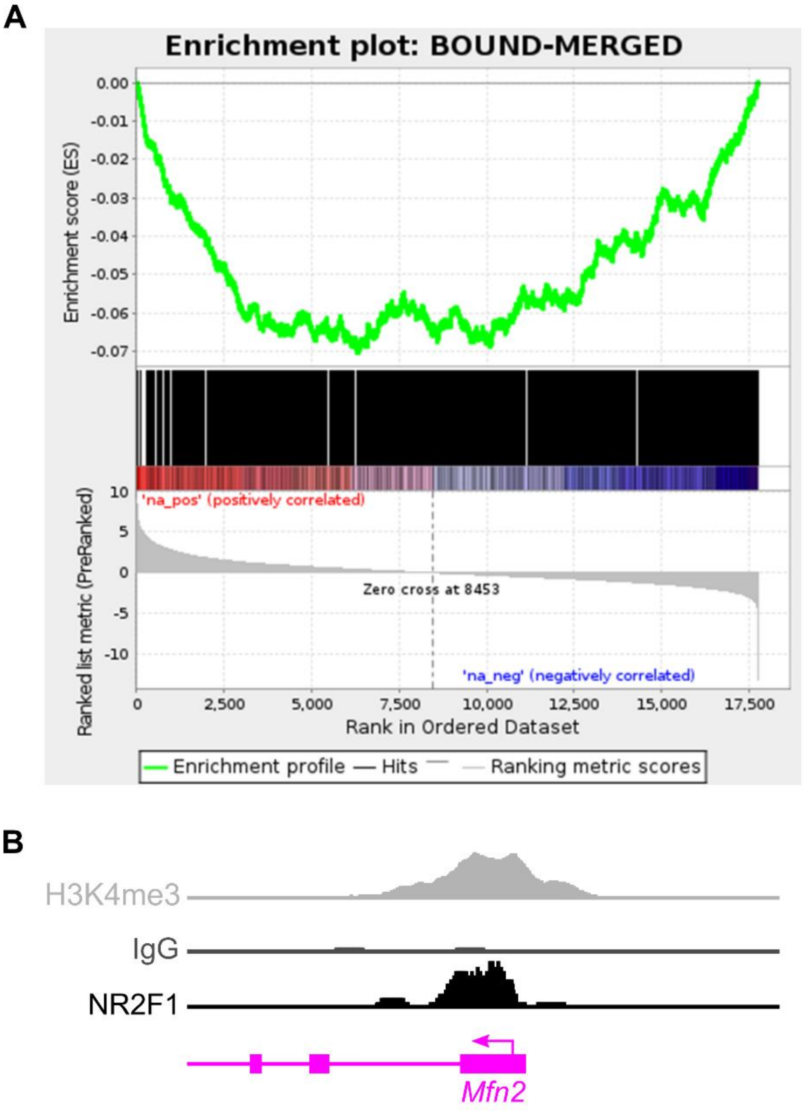

Fig. S4. Related to Figure 4 and 5. A. Gene set enrichment analysis (GSEA) enrichment plot showing downregulation of Nr2f1 targets in adult mice constitutively heterozygous for *Nr2f1*. The genes are ranked on the x axis according to the differential expression metric computed by Chen et al. 2020. The bottom panel shows the differential expression metric, the middle panel shows genes belonging to the gene set considered (NR2F1 targets identified though ChIP-seq) as black tics. The green curve in the top panel displays the running sum of the GSEA enrichment score (Normalized Enrichment Score < -2.9, FDR < 0.001). **B.** Sequence tag accumulation of the NR2F1 binding sites identified by ChIP-seq around the TSS region of the *Mfn2* gene and the concomitant accumulation of H3K4me3 are shown.

Disease Models & Mechanisms • Supplementary information

Table S1.

Click here to download Table S1

Table S2. List of the primary antibodies used for immunofluorescence

Antigen name	Host	Dilution	Source	Catalogue number
**Primary antibodies**				
BrdU	Rat	1:3000	AbD Serotec	OBT0030CX
COUP-TFI/NR2F1	Rabbit	1:1000	Abcam	ab181137
COUP-TFI/NR2F1	Rabbit	1:1000	Studer's lab	-
DCX	Goat	1:1500	Santa Cruz Biotechnology	Sc-8066
GFAP	Goat	1:2000	Abcam	ab53554
GFP	Chicken	1:1000	AvesLab	GFP-1020
LAMP1	Rat	1:300	Santa Cruz Biotechnology	Sc-19992
MFN2	Rabbit	1:500	Immunological Science	MAB-94608
NeuN	Mouse	1:1000	Chemicon	MAB377
OPA1	Rabbit	1:800	Cell Signalling	mAb#80471
ETC/OxPhos Mix	Mouse	1:500	Abcam	ab110413
PROX1	Rabbit	1:1000	Immunological Science	AB-84319
RFP	Rabbit	1:1000	Rockland	600-401-379
ZIF268	Rabbit	1:500	Santa Cruz Biotechnology	sc-189

Table S3. List of the secondary antibodies used for immunofluorescence

Antigen name	Host	Dilution	Source	Catalogue number
**Secondary antibodies**				
AlexaFluor488 Anti-Ck	Donkey	1:400	Jackson ImmunoResearch	703-545-155
AlexaFluor488 Anti-Gt	Donkey	1:400	Jackson ImmunoResearch	705-545-147
AlexaFluor488 Anti-Ms	Donkey	1:400	Jackson ImmunoResearch	715-545-151
AlexaFluor647 Anti-Gt	Donkey	1:600	Jackson ImmunoResearch	705-605-147
AlexaFluor647 Anti-Ms	Donkey	1:600	Jackson ImmunoResearch	715-605-151
AlexaFluor647 Anti-Rb	Donkey	1:600	Jackson ImmunoResearch	711-605-152
AlexaFluor647 Anti-Rat	Donkey	1:600	Jackson ImmunoResearch	712-605-153
Cy3 Anti-Ms	Donkey	1:800	Jackson ImmunoResearch	715-165-151
Cy3 Anti-Gt	Donkey	1:800	Jackson ImmunoResearch	705-165-147
Cy3 Anti-Rb	Donkey	1:800	Jackson ImmunoResearch	711-165-152
Cy3 Anti-Rat	Donkey	1:800	Jackson ImmunoResearch	712-165-153

Table S4. List of the primary antibodies used for WB analysis

Antigen name	Host	Dilution	Source	Catalogue number
**Primary antibodies**				
MFN2	Mouse	1:1000	Santa Cruz Biotechnology	sc-100560
OPA1	Rabbit	1:1000	Cell Signalling	mAb#80471
ETC/OxPhos	Mouse	1:1500	Abcam	ab110413
VDAC	Rabbit	1:1000	Cell Signalling	mAb#4661

Table S5. List of the secondary antibodies used for WB analysis

Antigen name	Host	Dilution	Source	Catalogue number
**Secondary antibodies**				
HRP Conjugated-anti-mouse	Goat	1:15.000	Cell Signalling	#7076
HRP Conjugated-anti-rabbit	Goat	1:15.000	Cell Signalling	#7074

Disease Models & Mechanisms • Supplementary information
